# *Epichloë* Endophytes Alter Inducible Indirect Defences in Host Grasses

**DOI:** 10.1371/journal.pone.0101331

**Published:** 2014-06-30

**Authors:** Tao Li, James D. Blande, Pedro E. Gundel, Marjo Helander, Kari Saikkonen

**Affiliations:** 1 Department of Environmental Science, University of Eastern Finland, Kuopio, Finland; 2 Plant Production Research, MTT Agrifood Research Finland, Jokioinen, Finland; 3 Department of Biology, University of Turku, Turku, Finland; Umeå Plant Science Centre, Umeå University, Sweden

## Abstract

*Epichloë* endophytes are common symbionts living asymptomatically in pooid grasses and may provide chemical defences against herbivorous insects. While the mechanisms underlying these fungal defences have been well studied, it remains unknown whether endophyte presence affects the host's own defences. We addressed this issue by examining variation in the impact of *Epichloë* on constitutive and herbivore-induced emissions of volatile organic compounds (VOC), a well-known indirect plant defence, between two grass species, *Schedonorus phoenix* (ex. *Festuca arundinacea*; tall fescue) and *Festuca pratensis* (meadow fescue). We found that feeding by a generalist aphid species, *Rhopalosiphum padi*, induced VOC emissions by uninfected plants of both grass species but to varying extents, while mechanical wounding failed to do so in both species after one day of damage. Interestingly, regardless of damage treatment, *Epichloë uncinata*-infected *F. pratensis* emitted significantly lower quantities of VOCs than their uninfected counterparts. In contrast, *Epichloë coenophiala*-infected *S. phoenix* did not differ from their uninfected counterparts in constitutive VOC emissions but tended to increase VOC emissions under intense aphid feeding. A multivariate analysis showed that endophyte status imposed stronger differences in VOC profiles of *F. pratensis* than damage treatment, while the reverse was true for *S. phoenix*. Additionally, both endophytes inhibited *R. padi* population growth as measured by aphid dry biomass, with the inhibition appearing greater in *E. uncinata*-infected *F. pratensis*. Our results suggest, not only that *Epichloë* endophytes may play important roles in mediating host VOC responses to herbivory, but also that the magnitude and direction of such responses may vary with the identity of the *Epichloë*–grass symbiosis. Whether *Epichloë*-mediated host VOC responses will eventually translate into effects on higher trophic levels merits future investigation.

## Introduction

Plants form intimate associations with a myriad of microorganisms, either detrimental or beneficial [1]–[5]. One of the most widely studied associations is the symbiotic defensive mutualism between Pööideae grasses and endophytic fungi of the genus um *Epichloë*, due to their significant impacts on insect and mammalian herbivores, particularly in agricultural pastoral systems [2]–[4], [6]–[8]. In exchange for shelter, nutrition and transmission via host seeds, the endophytes may increase host growth and reproduction, stress tolerance and herbivore and pathogen resistance (e.g. [1], [9]). Moreover, they may also have community-wide impacts by affecting secondary consumers and altering interplant competition [4], [10]–[12].

The endophyte-conferred herbivore resistance is often attributed to the direct induction of biologically active alkaloids by the endophyte, which may adversely affect herbivore performance [9], [13]–[16]. However, the alkaloid profiles and concentrations may vary considerably among grass-endophyte systems and environmental conditions, leading to no effects, or even positive effects, on herbivores [7], [15]–[19]. These variable effects indicate that apart from endophyte-conferred alkaloid defence, additional as-yet-undiscovered mechanisms such as endophyte-mediated changes in host defence chemistry are likely to be implicated in complex endophyte-host-insect interactions. In plant-microbe interactions some beneficial microbes (e.g. mycorrhizal fungi and plant growth-promoting rhizobacteria) have been shown to affect herbivore performance by inducing their host plant's own defences [20], [21]. Yet, this has been largely disregarded in previous studies concerning grass-endophyte symbioses [16], [22].

Among the multitude of plant responses to biotic and abiotic stimuli is the release of complex blends of volatile organic compounds (VOCs) that consist mainly of terpenoids, fatty acid derivatives and phenylpropanoids. Plant VOCs play an important ecological role in shaping the assemblage of, and interactions between, organisms within a plant's community [23]–[27]. For example, herbivorous insects rely, to varying extents, on plant VOCs as olfactory cues to recognize host plants and avoid non-host plants [23], [24]. On the other hand, herbivore attack elicits both local and systemic emission of plant VOCs, which in turn may protect plants from further damage either directly through deterring or repelling herbivores or indirectly through attracting natural enemies of herbivores [24], [25]. Interestingly, accumulating evidence has revealed that colonization by plant growth-promoting rhizobacteria or arbuscular mycorrhizal fungi can also modify constitutive or herbivore-induced VOC emissions of colonized plants and thereby alter VOC-mediated multitrophic interactions [28]–[36], although often in unpredictable ways.

Little is known about the effects of fungal endophytes on VOC emissions of host plants and the cascading effects on the behaviour of insects at different trophic levels. The few studies on endophyte-induced alteration of VOC blends have found that VOC emissions may be enhanced [37], reduced [38], or unchanged by endophyte presence [20] depending on plant and endophyte species. However, these studies mainly involve horizontally transmitted fungal endophytes and constitutive VOC emissions. In contrast to horizontally transmitted endophytes, vertically transmitted fungal *Epichloë* endophytes form a life-long and heritable symbiosis with their host grasses (e.g. [1], [9]), and hence may alter the host's chemistry differently. Furthermore, systemic endophytes have long dominated the literature presumably because of their agronomic impact (e.g. [2], [9]), but their effects on constitutive and herbivore-induced VOC emissions have been poorly explored. The only information on this comes from an early study of *Epichloë coenophiala*—infected tall fescue (*Schedonorus phoenix*) [39] and a very recent study of *Epichloë festucae* var. *lolii*-infected perennial ryegrass (*Lolium perenne*) [40], both demonstrating the potential effect of *Epichloë* endophytes on host's VOC release but failing to determine the impact of herbivory. Regardless of which group they belong to, elucidating the endophyte effects on both constitutive and herbivore-induced VOC emissions in endophyte-grass interactions would not only help to understand the variable endophyte effects on herbivory, but also to evaluate the potential of endophytes as biological control tools.

We investigated whether and how *Epichloë* endophytes altered constitutive and herbivore-induced VOC emissions in two fescue species, tall fescue (symbiotic with *Epichloë coenophiala*) and meadow fescue (*Festuca pratensis*, symbiotic with *Epichloë uncinata*). To examine herbivore-induced VOC emissions, we used a generalist aphid species, *Rhopalosiphum padi* (bird cherry-oat aphid), a common pest of grasses that has been extensively used as a model phloem feeder in grass endophyte research (e.g. [9]). Specifically, we addressed the following questions: (1) Does aphid feeding induce VOC emissions in grasses? (2) Does *Epichloë* infection modify VOC emissions by its host? (3) Is there any variation in these responses of the two grass species? We discuss how VOC emissions by the host could interact with endophyte-conferred defence.

## Materials and Methods

### Ethics statement

This study was conducted in the laboratory and did not involve any endangered or protected species; the insect species used in this study is a serious cereal pest worldwide, including Finland. Hence, no specific ethical approval was required for this study.

### Plants, endophytes and Insects

Seeds of naturally endophyte infected (E+) and endophyte-free (E-) tall fescue (*Schedonorus phoenix*) cultivar ‘Kentucky 31′ and meadow fescue (*Festuca pratensis*) cultivar ‘Kasper’ were collected from experimental fields in the University of Turku Ruissalo Botanical Garden. For tall fescue, in addition to E+ and E- plants, manipulatively endophyte-free (ME-) plants, which were obtained from E+ plant using heat treatment, were included to separate the effects of endophyte infection from plant responses. Because host plants of different endophyte status are from a single cultivar, genetic variation and its impact on plant defence response might be limited compared to wild plants. The infection status of the plants was verified by growing out the fungus from surface sterilized grass leaf cuttings plated on potato dextrose agar (PDA) in Petri dishes [41], [42]. Plants were grown individually in 12-cm-diameter plastic pots filled with a standard potting soil in a greenhouse [photosynthetically active radiation (PAR) at canopy level ca. 300 µmol m^−2^ s^−1^]. Approximately 60 days after seedling transplantation, plants were transferred to a climate-controlled room (18–24°C, 70% RH, L16:D8 photoperiod, and ca. 250 µmol m^−2^ s^−1^ PAR) in the laboratory, where all experiments were conducted.

*Rhopalosiphum padi* were obtained from a colony at the Department of Ecology at the Swedish University of Agricultural Sciences in Uppsala, Sweden, and were reared on barley in a climate-controlled room (16L:8D, 18–24°C, and 70% RH).

### Plant treatment

Experiments for both fescue species followed a full-factorial experimental design with two factors, endophyte infection and damage inflicted either by aphid infestation or mechanical wounding. Before the start of the experiments, a total of 18 plants without visible damage were selected from each endophyte status (E-, ME- and E+ for tall fescue; E- and E+ for meadow fescue) and divided into six groups according to plant size, with plants in each group characterized by similar size. The three plants within each group were then randomly subjected to the following three treatments: 1) aphid infestation (A): plants were infested by placing five barley leaf segments containing a total of 50 mixed-instar nymphs and apterae between tillers and removing these segments after all aphids had moved onto the plants; 2) wounding (W): plants were mechanically damaged by first cutting ca. 4 cm off every leaf tip with scissors and then squeezing the remaining leaf blade eight times with forceps; and 3) control (C): control plants received no damage. Mechanical wounding was meant to mimic the damage caused by animal grazing and trampling. In total, there were nine and six endophyte × damage treatments for tall and meadow fescue, respectively, each containing six plants. To avoid aphids moving to neighbouring plants, all plants including those without aphids were placed in screened cages with cage positions rotated daily to control for any differences in light or temperature conditions.

VOCs from wounded and aphid-infested plants were collected at different times following treatment. Mechanical damage typically elicits rapid release of VOCs, particularly green leaf volatiles (GLVs; e.g. [43]). Consistent with this, we found in a preliminary study with red fescue (*Festuca rubra*) that 40 min after mechanical wounding similar to that in this study, GLV emissions were several hundred-fold higher compared to pre-damage emissions (Figure S1). After one day, GLV emissions remained substantially higher than pre-damage emissions; however, an induction of few terpenoids (e.g. β-ocimene) manifested itself. Therefore, we collected VOCs from mechanically wounded plants one day after wounding to capture responses of both GLV and terpenoids compounds. For meadow fescue, VOC collection was also performed at six days post-wounding. However, since VOC responses induced by phloem feeders – which inflict minimal tissue damage – proceeds slowly with a delay of several days [34], [44], we collected VOCs from aphid-infested plants at 6 and 12 days after infestation. This timing was chosen according to the time courses of aphid-induced VOC induction reported in the literature [34], [44].

After the last VOC sampling, the aboveground plant parts, which had been enclosed during VOC collection, were harvested, oven-dried and weighed. Owing to high numbers of aphid offspring on infested plants, in particularly on infested E- plants, total dry weight of aphids per plant (including dead ones present on the plants) rather than aphid number was determined and used as a proxy for aphid propagation and growth to assess the endophyte effects on aphid performance.

### VOC collection and analysis

VOC collection was conducted as in Li *et al*. [27]. In brief, the pots plus soil were carefully wrapped with aluminium foil to prevent contamination with soil-derived volatiles. The aerial part of each potted plant was then enclosed in a polyethylene terephthalate (PET) bag and sealed with a plastic-coated wire. Charcoal filter purified air was pushed through Teflon tubing into each bag (230 ml min^−1^) and pulled out by a vacuum pump (200 ml min^−1^) through a stainless steel trap packed with 150 mg of Tenax TA and 150 mg of Carbopack B (Markes International, Llantrisant, RCT, UK). VOCs were collected for 1 h and simultaneously from plants of different treatments; periodic collections of VOCs from empty PET bags were also made. Aphids were kept on infested plants during collection since the presence of aphids has been shown to contribute little if anything to the VOC blends emitted by the plant-aphid complex [34], [44]. Furthermore, it is likely that removing aphids from plants will result in a degree of mechanical damage to the plant and may result in aphids emitting their alarm pheromones.

VOC samples were analyzed by GC-MS (Hewlett-Packard GC 6890; MSD 5973; Wilmington, DE, USA). Trapped volatiles were desorbed with a thermal desorption unit (ATD400; Perkin Elmer, Waltham, MA, USA) at 250°C for 10 min, focused at −10°C on a cold trap and transferred onto an HP-5 capillary column (50 m×0.2 mm; film thickness 0.5 µm) with helium as the carrier gas (1.2 ml min^−1^). The column temperature was initially held at 40°C for 1 min, then ramped to 210°C at 5°C min^−1^, and finally to 250°C at 20°C min^−1^. Individual VOCs were tentatively identified by comparing mass spectra with those in NIST and Wiley spectral libraries and verified by chromatography with authentic standards when available. Although over 50 prominent peaks could be detected, only those that were found consistently higher in the samples than in the blanks were considered in further analyses, allowing identification of 17 compounds in both grass species. For quantification, peak areas of characteristic quantifier ions were integrated and the amount of each compound was calculated based on external calibration curves generated with authentic standards. For compounds whose reference standards were not available, quantification was assessed relative to the external standard 1-chlorooctane. Emission rates were presented in nanograms per gram dry weight per hour (ng g^−1^DW h^−1^).

### Statistical analysis

To analyse the main effects of endophyte (E- versus E+), aphid (C versus A), sampling date (6 days versus 12 days post-infestation) and their interaction on emissions of total and single VOCs, we used a linear mixed model (LMM) for each grass species, with endophyte and aphid as the between-subjects factors and time as the within-subjects factor. Since *R. padi* aphids performed badly on E+ plants relative to E- plants (see ‘Results’), E+ plants may experience less leaf damage over the infestation period. To account for any potential effects of differential aphid damage on VOC emissions, we used the total aphid dry weight per plant as an indicator of the extent of plant damage, and re-analyzed aphid-induced VOCs by including aphid dry weight as a covariate in the LMM. In this model, endophyte, sampling date and their interaction were fixed factors. To examine endophyte by wounding effects, we performed two-way ANOVA. Effects of endophyte on aphid dry weight and effects of endophyte by aphid on plant dry mass were analysed by one-way and two-way ANOVA, respectively. Data were log transformed [log(X+1)] to meet normality and homoscedasticity. All analyses were performed using the statistical package SPSS 19.0 for windows.

To visualize differences in VOC blends of differently treated plants, data were also analysed with Partial Least Projection to Latent Structures-Discriminant Analysis (PLS-DA) (SIMCA-P11.0; Umetrics, Umeå, Sweden). To preprocess data, emission rates of individual VOCs were normalized [log(X+1)], mean-centred and scaled to unit variance. The number of significant PLS components was determined by cross validation [45]. This method allows not only for visualization of high dimensional data in score plots, but also for identification of variables (i.e. volatile compounds) that are important for the differences in complex VOC blends among treatments. In general, variables with the Variable Importance in the Projection (VIP) scores larger than 1 are considered most influential for the model.

## Results

### VOC emissions by tall fescue

**Figure 1:**
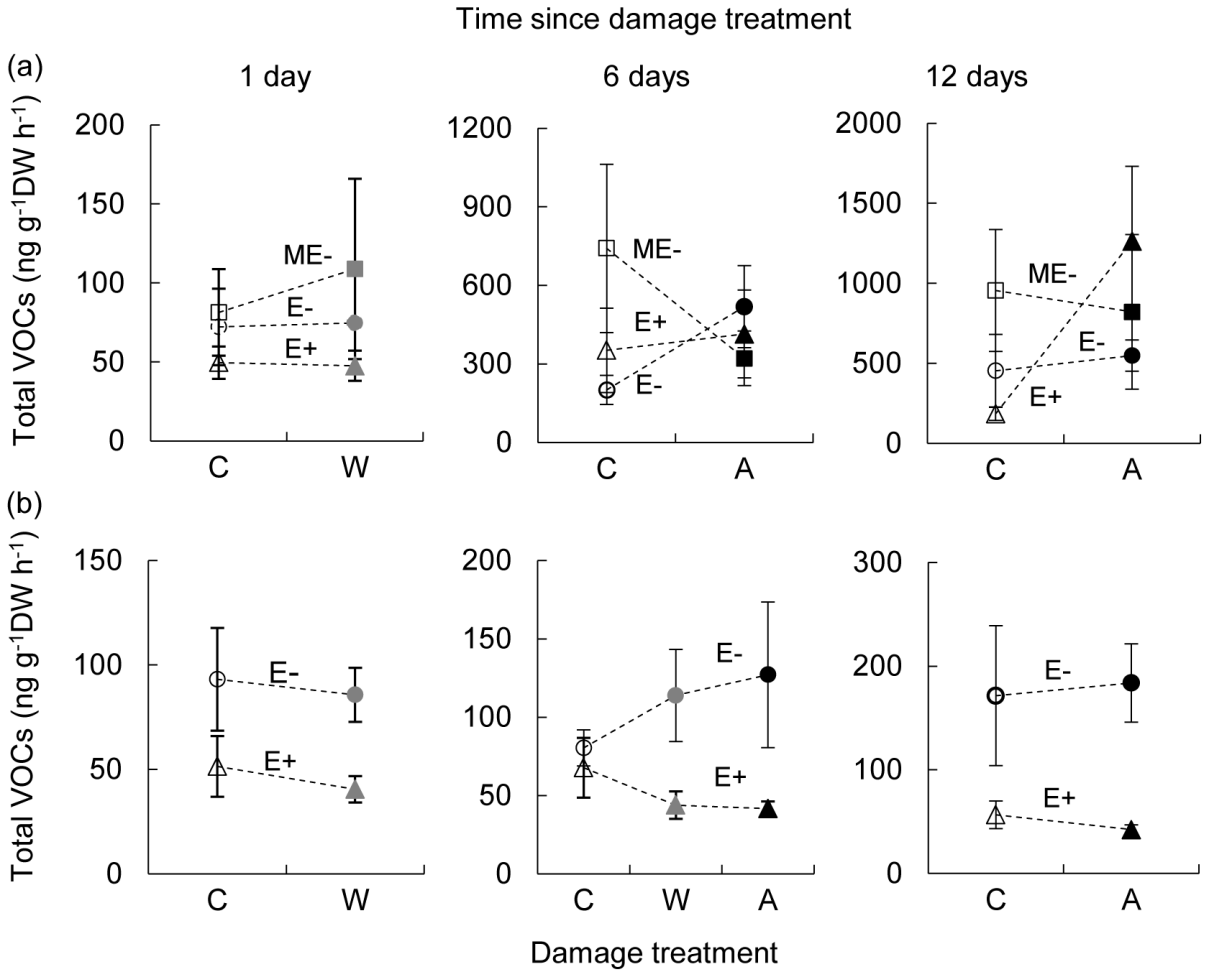
Total VOC emissions (mean ±1SE) from naturally endophyte free (E-; circles) vs infected (E+; triangles) tall fescue (a) and meadow fescue (b) in response to aphid or mechanical damage. C: untreated control; W: mechanical wounding; A: aphid infestation. For comparison, manipulatively endophyte free tall fescue (ME-; squares) was included. All damage treatments were initiated at the same time, then VOC collections conducted at 1 day after mechanical wounding, or at 6 and 12 days after aphid addition. Statistical details are shown in tables 1 and 2.

**Figure 2:**
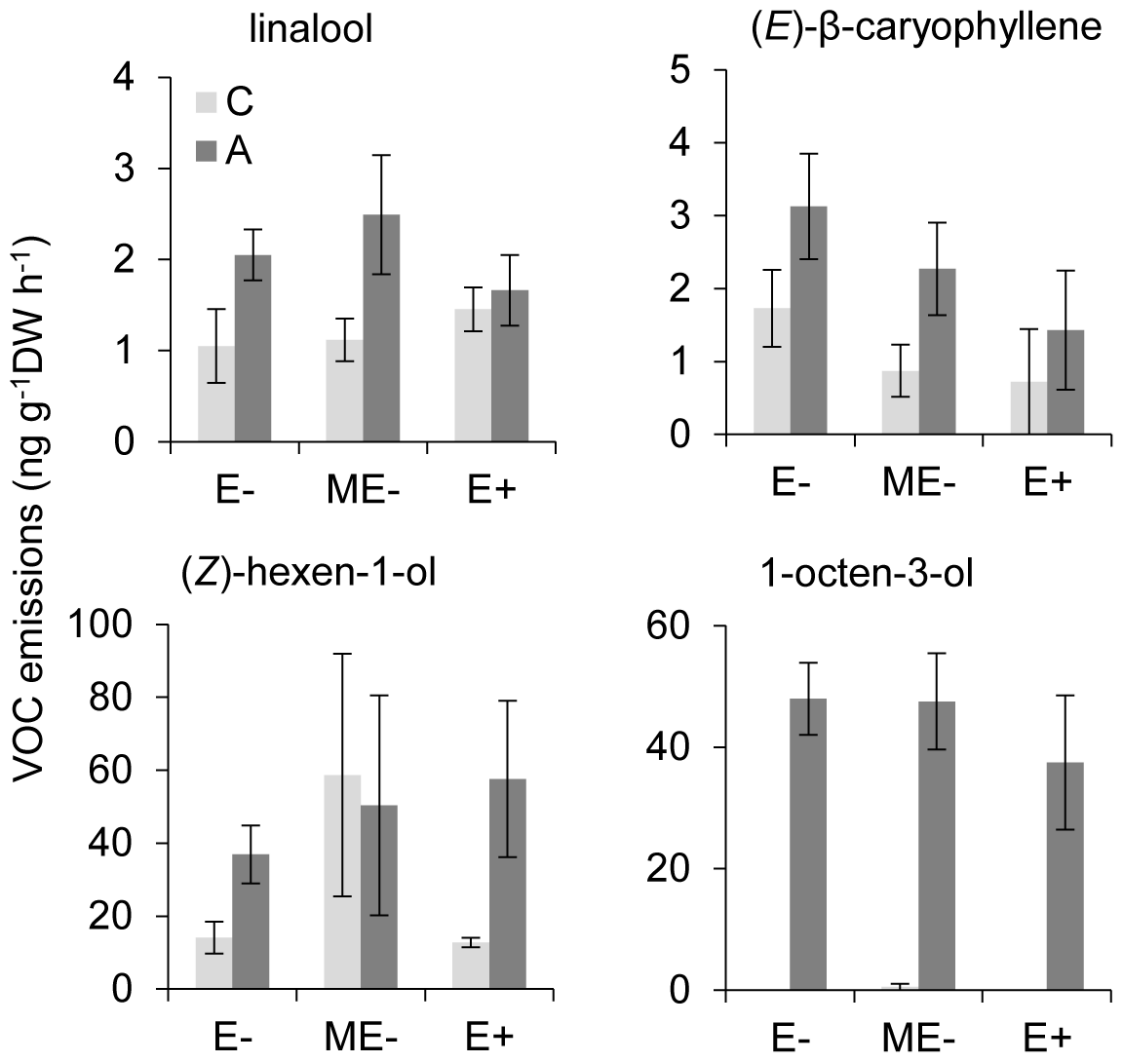
Individual VOCs of tall fescue whose release changed significantly in response to endophyte infection and 12 days of aphid feeding. E-: naturally endophyte free; E+: naturally endophyte infected; ME-: manipulatively endophyte free; C: untreated control; A: aphid damage. Statistical details are shown table S1.

There was no significant effect of endophytes (LMM; *F*_(2, 59.99)_  = 0.68, *P* = 0.512) and aphids (*F*_(1, 59.99)_  = 2.23, *P* = 0.155) on total VOC emissions, though aphid-infested E+ plants appeared to increase total emissions after 12 days of feeding (Figure 1a; Tables 1, S1 and S2). Regarding individual compounds, endophyte presence decreased emissions of (*Z*)-β-ocimene (*P* = 0.059) and (*E*)-β-caryophyllene (*P* = 0.005) irrespective of feeding treatment, whereas endophyte removal increased emissions of α-pinene (*P*<0.001), an unknown monoterpene (*P* = 0.010) and methyl salicylate (*P* = 0.043). After controlling for possible effects of differential levels of leaf damage, similar results were observed for the effects of endophyte presence on VOC emissions from aphid-infested plants (Table S3). Aphid feeding induced differential responses of several compounds depending on endophyte status and feeding duration. At 12 days post-feeding, for example, infested E+ plants emitted higher amounts of (*Z*)-3-hexen-1-ol (*P* = 0.073) and (*Z*)-3-hexen-1-ol acetate (*P* = 0.093) than control E+ plants, while infested E- plants emitted substantially more linalool (*P* = 0.016), (*E*)-β-caryophyllene (*P* = 0.088) and (*Z*)-3-hexen-1-ol (*P* = 0.038) than control E- plants (Figure 2; Table S1). The most notable aphid effect was the de novo induction of 1-octen-3-ol (*P*<0.001), which was released exclusively from infested plants independently of endophyte status and in significantly higher amounts at 12 days after aphid addition than at 6 days (*P*<0.001). There was no significant effect of mechanical damage on either total or individual VOC compounds (Table S4).

**Table 1:** Summary of LMM results showing the level of significance for the effect of endophyte (E), aphid (A) and sampling time (T) on VOC emissions of tall fescue.

		E		A		T		Interaction
Compound	*ndf*	*F*	*P*	*F*	*P*	*F*	*P*	*P*
Terpenoids								
α-pinene	56.89	10.92	**<0.001**	1.46	0.231	100.09	**<0.001**	
6-methyl-5-hepten-2-one†	57.39	0.38	0.683	1.02	0.317	0.14	0.708	
β-myrcene	53.21	0.84	0.437	1.21	0.277	2.32	0.133	E x A: **0.010**
β-pinene	36.72	0.31	0.738	0.00	0.970	3.25	**0.080**	
δ-carene	52.75	0.86	0.427	2.72	0.105	1.63	0.208	
(*Z*)-β-ocimene†	58.65	2.97	**0.059**	4.47	**0.039**	0.04	0.833	
d-limonene	49.70	0.69	0.506	0.00	0.997	1.39	0.244	E x A: **0.069**
β-phellandrene†	47.08	0.22	0.806	0.09	0.764	5.71	**0.021**	
(*E*)-β-ocimene	59.54	1.61	0.208	2.97	**0.090**	0.10	0.759	
α-terpinolene	55.75	0.23	0.796	0.04	0.838	1.96	0.167	
linalool*	57.57	1.15	0.325	10.23	**0.002**	1.46	0.232	
Unkown monoterpene†	59.93	5.03	**0.010**	1.12	0.295	6.60	**0.013**	
(*E*)*-*β*-*caryophylene*	56.06	5.75	**0.005**	5.57	**0.022**	0.60	0.442	
Total Terpenoids	59.16	2.75	**0.072**	2.19	0.144	1.58	0.213	
Green leaf volatiles (GLV)								
(Z)-3-hexen-1-ol*	60.00	0.72	0.491	2.76	0.102	0.02	0.899	E x A: **0.061**
(Z)-3-hexen-1-ol acetate	59.96	0.76	0.472	0.92	0.341	2.35	0.130	
Total GLV	60.00	0.74	0.482	1.13	0.292	2.01	0.161	
Other compounds								
1-octen-3-ol*	42.60	1.43	0.250	282.62	**<0.001**	44.21	**<0.001**	A x T: **<0.001**
methyl salicylate	57.75	3.32	**0.043**	5.67	**0.021**	0.06	0.811	A x T: **0.043**
Total VOCs	59.99	0.68	0.512	2.07	0.155	2.23	0.142	

Bold numbers indicated effects with *P* values less than 0.1 as determined by LMM. *ndf* represents the **n**umerator **d**egrees of **f**reedom; the **d**enominator **d**egrees of **f**reedom (*ddf*) were 2 for E and 1 for A and T. For details on emission rates see Table S1 and S2.

*Compounds that were identified by PLS-DA analysis as most influential for separation of individual treatments at 12 days post aphid feeding.

† Compounds tentatively identified.

### VOC emissions by meadow fescue

**Figure 3:**
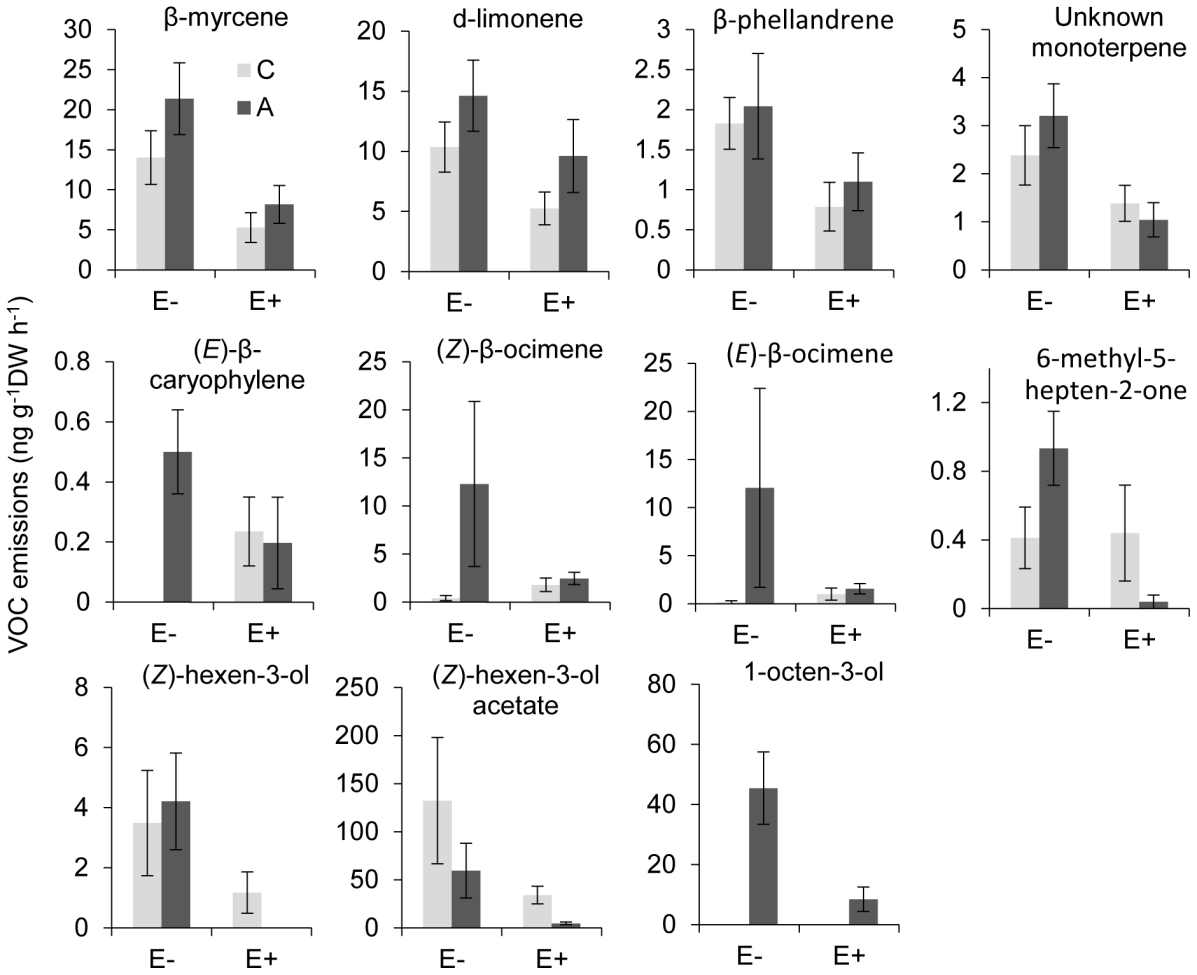
Individual VOCs of meadow fescue which changed significantly in response to endophyte infection and 12 days of aphid feeding. E-: naturally endophyte free; E+: naturally endophyte infected; C: untreated control; A: aphid damage. Statistical details are shown table S5.

Unlike tall fescue, meadow fescue exhibited significantly reduced emissions of total VOCs in the presence of endophyte (LMM; *F*_(1, 36.99)_  = 17.47, *P*<0.001; Figure 1b; Tables 2, S5 and S6) as well as emission of nine components, including 6-methyl-5-hepten-2-one (*P* = 0.012), β-myrcene (*P*<0.001), d-limonene (*P*<0.001), β-phellandrene (*P*<0.001), α-terpinolene (*P* = 0.001), (*Z*)-3-hexen-1-ol (*P* = 0.006), (*Z*)-3-hexen-1-ol acetate (*P* = 0.002), 1-octen-3-ol (*P* = 0.002), and the same unknown monoterpene (*P*<0.001) detected in tall fescue. These emission reductions were consistently observed at all three sampling times regardless of whether plants were damaged or not. Moreover, reduced emissions of VOCs by aphid-infested E+ plants were still evident after adjusting for potential effects of differential leaf damage (Table S7). Aphid infestation did not affect total emissions (*P* = 0.755) either at 6 or 12 days of infestation, but induced emissions of β-myrcene (*P* = 0.036), (*Z*)-β-ocimene (*P* = 0.002), d-limonene (*P* = 0.005), (*E*)-β-ocimene (*P* = 0.025), (*E*)-β-caryophyllene (*P* = 0.063) and 1-octen-3-ol (*P*<0.001) at 12 days whilst depressing (*Z*)-3-hexen-1-ol acetate emission (*P* = 0.008) (Figure 3; Table S5). Again, the most pronounced effect of aphid feeding was the de novo induction of 1-octen-3-ol as observed in tall fescue. Additionally, there was a significant or marginally significant interaction between endophytes and aphids on emissions of 6-methyl-5-hepten-2-one (*P* = 0.051), (*Z*)-3-hexen-1-ol (*P* = 0.070), (*Z*)-3-hexen-1-ol acetate (*P* = 0.079) and 1-octen-3-ol (*P* = 0.002), with infested E+ plants emitting less of these compounds than plants in any other treatment. Neither mechanical damage nor its interaction with endophytes affected VOC emissions at 1 day following damage (Table S8).

**Table 2:** Summary of LMM results showing the level of significance for the effect of endophyte (E), aphid (A) and sampling time (T) on VOC emissions of meadow fescue.

		E		A		T		Interaction
Compound	*ndf*	*F*	*P*	*F*	*P*	*F*	*P*	*P*
Terpenoids								
α-pinene	29.60	0.02	0.879	0.50	0.484	25.59	**<0.001**	
6-methyl-5-hepten-2-one^†^ *	38.88	6.98	**0.012**	0.13	0.720	5.40	**0.025**	E x A: **0.051**
β-myrcene*	30.14	19.77	**<0.001**	4.79	**0.036**	1.33	0.259	
β-pinene	33.42	0.20	0.659	0.87	0.356	7.70	**0.009**	
δ-carene	39.56	0.07	0.796	0.83	0.369	11.66	**0.001**	
(*Z*)-β.ocimene^†^ *	35.66	0.00	0.961	11.42	**0.002**	1.36	0.250	
d-limonene*	39.24	17.98	**<0.001**	8.97	**0.005**	9.38	**0.004**	
β-phellandrene^†^	39.86	15.75	**<0.001**	0.25	0.621	0.10	0.752	
(*E*)*-*β-ocimene*	35.52	0.37	0.548	5.46	**0.025**	0.96	0.334	
α-terpinolene	39.62	12.47	**0.001**	1.43	0.239	29.77	**<0.001**	
linalool	37.38	1.77	0.192	0.90	0.349	11.45	**0.002**	
Unkown monoterpene^†^ *	37.64	14.57	**<0.001**	0.99	0.326	1.17	0.286	
(*E*)*-*β-caryophylene*	35.23	0.89	0.353	3.69	**0.063**	0.03	0.856	E x A x T: **0.073**
Total Terpenoids	35.78	16.14	**<0.001**	12.22	**0.001**	0.01	0.913	
Green leaf volatiles (GLV)								
(*Z*)-3-hexen-1-ol*	39.46	8.35	**0.006**	1.08	0.305	0.17	0.685	E x A: **0.070**
(*Z*)-3-hexen-1-ol acetate*	38.73	10.95	**0.002**	7.77	**0.008**	0.01	0.908	E x A: **0.079**
Total GLV	38.72	11.42	**0.002**	7.56	**0.009**	0.01	0.903	E x A: **0.069**
Other compounds								
1-octen-3-ol*	29.31	11.83	**0.002**	51.63	**<0.001**	39.63	**<0.001**	All: **<0.002**
methyl salicylate	38.16	3.41	**0.072**	0.23	0.632	0.25	0.618	
Total VOC	36.99	17.47	**0.000**	0.10	0.755	0.73	0.397	

* and † as in Table 1. See Table S5 and S6 for details on emission rates.

### Visualization of differences in VOC profiles among treatments

**Figure 4:**
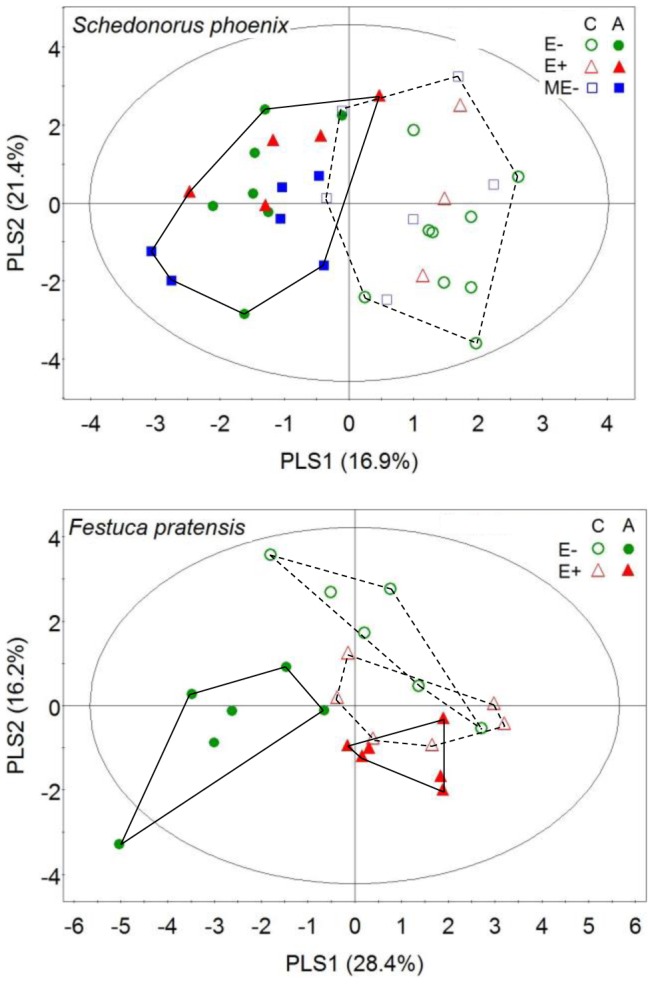
PLS-DA plots based on comparisons among VOC blends emitted by differently treated plants at 12 days after aphid addition. E-: naturally endophyte free; E+: naturally endophyte infected; ME-: manipulatively endophyte free; C: control; A: aphid feeding. For tall fescue (upper panel), a clear separation was seen between control and infested plants, which clustered mainly on the right and left side of the plot, respectively, whereas plants of different endophyte status within each damage treatment largely overlapped. For meadow fescue (lower panel), E- and E+ plants grouped separately while overlapping somewhat, with the strongest separation occurring between infested E- plants and any other treatment. Statistical details concerning compounds responsible for the clustering are given in tables S1 and S5.

For tall fescue VOC profiles of aphid-infested plants were distinctly different from control plants and the difference became more apparent at 12 days post-infestation (Figure 4) than at 6 days (Figure S2), whereas the profiles of ME-, E- and E+ plants were relatively similar to each other. In contrast, in meadow fescue a clear distinction between VOC blends of E- and E+ plants could be depicted at all three sampling days although a small overlap was seen (Figures 4, S2 and S3). VOC blends from control and aphid-infested plants grouped separately from each other only at 12 days after infestation (Figure 4), with the most pronounced separation occurring between infested E- plants and plants in all other treatments. In both species, wound-induced VOC blends were not separated from constitutive VOC blends (Figure S3).

In both species, the compounds that contributed most strongly to the differences among VOC blends of differently treated plants were found to be the same compounds that had been demonstrated above to be significantly induced by endophytes, aphids and/or their interaction (Tables 1 and 2). Among them, 1-octen-3-ol had the strongest discriminatory power in differentiating infested and control plants.

### Aphid and plant growth

Endophyte presence reduced aphid performance as aphid colonies grown on E+ plants had much lower dry weights than on E- plants for both tall (*F*_(2, 18)_  = 12.19, *P*<0.001) and meadow fescue (*F*_(1, 12)_  = 52.37, *P*<0.001; Figure S4). Interestingly, in tall fescue aphids performed better on ME- plants than on E+ plants but not as well as on E- plants, and ME- plants had lower dry mass than both E- and E+ plants (Figure S5). This suggests that either endophyte removal or heat treatment to remove endophytes or both may hinder plant growth and aphid performance. Additionally, short-term aphid infestation did not influence plant growth for either tall (*F*_(2, 54)_  = 0.92, *P* = 0.406) or meadow fescue (*F*_(2, 36)_  = 0.05, *P* = 0.955; Figure S5).

## Discussion

Our results reveal that feeding by the generalist aphid *R. padi* induces VOC emissions in tall and meadow fescue that are known to serve in plant direct and indirect defences against herbivore attack. Most importantly, we demonstrate for the first time that *Epichloë* endophytes are capable of modifying both constitutive and aphid-induced VOC emissions of host grasses. Moreover, the two studied fescues differ substantially in VOC emission patterns in response to aphid infestation and endophyte infection.

Although intensively characterized in a range of plant species (e.g. [24]), VOC profiles have rarely been studied in *Festuca*, particularly in response to herbivory. The only relevant study involves induced VOC emissions by tall fescue upon exogenous application of jasmonic acid (JA) [39], a phytohormone widely used to mimic induced defence responses to leaf-chewing herbivores. Our results therefore expand upon previous findings, showing clear induction of VOC emissions from tall fescue, and also from meadow fescue, in response to the phloem-sucking herbivore *R. padi*. Compared to JA treatment, which increased amounts of several constitutive VOCs [39], we found that aphid feeding on tall fescue enhanced emissions of fewer components. This comes as no surprise given that leaf-chewers (or JA application) and phloem-suckers activate JA and salicylic acid (SA) signaling pathways, respectively and that these two pathways often antagonize each other and elicit emissions of a different set of volatile compounds [16], [26]. Additionally, phloem-suckers, which usually cause limited cell damage, do not elicit volatile responses as strongly as leaf-chewers [34], [44]. Several compounds, including two β-ocimene isomers, were induced by aphid feeding in meadow fescue but not in tall fescue, suggesting that meadow fescue is more responsive than tall fescue when challenged by aphids. However, there was some commonality in the induced VOCs, most notably 1-octen-3-ol, which was the only de novo synthesized compound induced by aphid feeding and positively related to aphid density. 1-octen-3-ol is emitted by squash plants infected with powdery mildew (*Podosphaera* sp.), and is a particular component of ‘mouldy odour’ and attractive to mycophagous twenty-spotted ladybird beetles (*Psyllobora vigintimaculata*) [46]. This compound is also an induced volatile of *Trifolium pratense* after damage by *Spodoptera littoralis* caterpillars [47]. Therefore, 1-octen-3-ol may serve as a component of multiple indirect defence responses.

Unlike herbivory, mechanical damage inflicted by single wounding events often triggers rapid VOC release, which then drops to the pretreatment level within a few hours (e.g. [43]). However, in some cases VOC responses induced by mechanical damage may require days to appear [48]. In line with these findings, our preliminary study with red fescue revealed a rapid rise and prolonged emission. Unexpectedly, in this study we did not detect induced VOC emissions in either tall or meadow fescue one day after mechanical wounding. However, we may have missed a rapid VOC response by providing a one-day recovery period following wounding before collecting VOCs. Nevertheless, these studies suggest that the speed and duration of VOC responses induced by mechanical damage may differ among plant species.

Interestingly, our study reveals that *Epichloë* endophytes affect both constitutive and aphid-induced VOC emissions of host grasses. In the absence of aphids, *E. uncinata*-infected meadow fescue had significant lower emission rates than uninfected counterparts, whereas *E. coenophiala*-infected tall fescue did not differ from uninfected plants. When subjected to aphid attack, endophyte-infected meadow fescue still released significantly lower amounts of VOCs, but infected tall fescue tended to increase VOC emissions after 12 days of continuous feeding. In particular, endophytes and aphids appeared to act synergistically to suppress production of the two dominant compounds (*Z*)-hexen-1-ol and (*Z*)-3-hexen-1-ol acetate in meadow fescue but promote their release in tall fescue. The differences in aphid-induced VOC release between endophyte-free and infected plants remained pronounced even after accounting for potential effects of different extents of damage, suggesting that endophyte-mediated changes in host VOC release might occur regardless of intensity of herbivory. One caveat of our study is that even though single cultivars were used (Kentucky 31 of tall fescue; Kasper of meadow fescue), host genotype was not strictly controlled and may have influenced VOC emissions alone or interactively with endophytes.

In line with our findings, an early study with tall fescue has shown that *E. coenophiala-* infected plants did not differ in constitutive VOC emissions from uninfected counterparts, but doubled (Z)-3-hexen-1-ol acetate emission in response to JA treatment while decreasing emissions of a few terpenoids such as (*E*)-β-ocimene [39]. However, a recent study of perennial ryegrass has found greater quantities of both constitutive and pathogen-elicited VOC emissions emitted by *E. festucae* var. *lolii*-infected plants than uninfected counterparts [40]. Together, all these studies suggest that endophyte-mediated adjustment of host VOC production may vary with the identity of *Epichloë*-grass symbiosis and the type of biotic stress.

Volatile compounds may act as plant defensive semiochemicals that disturb herbivore settlement and proliferation and/or recruit herbivores' natural enemies [23]–. For example, studies on plant-aphid-parasitoid interactions have revealed that 6-methyl-5-hepten-2-one, (*Z*)-3-hexen-1-ol, (Z)-3-hexen-1-ol acetate, (*E*)-β-caryophyllene and (*E*)-β-ocimene [49]–[52] can attract aphid parasitoids and linalool can directly repel aphids [50], [53], while methyl salicylate seemingly acts in both ways [52], [54], [55]. In previous and current studies, grass hosts have been found to change emission patterns of some of these compounds in response to *Epichloë* infection either alone or in conjunction with biotic stress. Therefore, it is likely that the altered volatile profiles may modify VOC-mediated multitrophic interactions. This hypothesis needs to be tested in future studies to disclose the ecological consequences of *Epichloë*-mediated change in host VOC emissions for herbivores, natural enemies, and thus plant fitness.

While our results show that *Epichloë*-mediated host VOC responses depend on endophyte and host species, the ecological and evolutionary processes that lead to such variation remain unclear and merit future study. As with the expression of constitutive and inducible plant defences, harbouring endophytes is costly because endophytes must procure all of their nutrients from the host, including precursors in the synthesis of secondary metabolites such as alkaloids (e.g. [1], [2]). Thus, evolutionary trade-offs may occur in *Epichloë*–grass symbioses. In other words, *Epichloë*-grass symbioses which have developed high levels of endophyte-derived resistance under natural or artificial selection may have evolved low levels of host defence, and vice versa. Our observation of *Epichloë* species related differences in host VOC responses partially support this idea. Specifically, the meadow fescue–*E. uncinata* symbiosis which has high endophyte-conferred constitutive defence [13], [14], [17], [56] released low amounts of VOCs by the host grass regardless of herbivore presence. By comparison, in the tall fescue–*E. coenophiala* symbiosis where *E. coenophiala* provides relatively low constitutive defence [13], [14], [17], [56], the host grass exhibited induced VOC responses in the presence of high aphid density.

In conclusion, our study has shown that *Epichloë* endophytes may modulate VOC responses of host grasses, with the sign and strength of such responses depending on the identity of the *Epichloë*–grass symbiosis. Our results illustrates the importance of assessing host plant volatiles and their impacts on herbivore host-searching behaviour to investigate alternative mechanistic links between *Epichloë* endophytes and herbivore responses. Given that both *Epichloë* endophytes and herbivores can manipulate their shared hosts in diverse ways and that endophyte-provided resistance to herbivores varies considerably among *Epichloë*–grass associations, *Epichloë*–mediated host VOC responses and their impacts on multitrophic interactions should be variable. Further studies with different *Epichloë*–grass associations would shed more light on endophyte-provided defence and its interaction with the host' own defence.

## Supporting Information

Figure S1 **Kinetics of VOC emissions from red fescue (** ***Festuca rubra*** **) following mechanical wounding.** (a) Total ion current (TIC) chromatograms of VOCs from a representative plant sample of red fescue before and after mechanical wounding. (b) Emissions (peak area ±SE; n = 4) of the dominant VOCs from red fescue. 1 =  (*Z*)-3-hexenal, 2 =  (*E*)-2-hexenal, 3 =  (*Z*)-3-hexen-1-ol, 4 =  (*Z*)-3-hexen-1-ol acetate, 5 =  (Z)-β-ocimene, 6 =  (*E*)-β-ocimene.
(TIF)

Figure S2 **PLS-DA plots of VOC blends emitted by differently treated plants at 6 days after aphid addition.** E-: naturally endophyte free; E+: naturally endophyte infected; ME-: manipulatively endophyte free; C: control; A: aphid feeding; W: mechanical wounding. For tall fescue (upper panel), a clear separation was seen between control and infested plants, whereas for meadow fescue (lower panel) the separation was mainly found between E- and E+ plants. Statistical details concerning compounds responsible for the clustering are given in table S2 and S6.
(TIF)

Figure S3 **PLS-DA plots of VOC blends emitted by differently treated plants at 1 day after mechanical wounding.** In tall fescue (upper panel) the strongest separation was observed between ME- plants and either of the E- and E+ plants, with the latter two largely overlapping. In meadow fescue (lower panel) E- and E+ plants, while overlapping somewhat, remained largely separated from each other (E-: naturally endophyte free; E+: naturally endophyte infected; ME-: manipulatively endophyte free; C: untreated control; W: mechanical wounding). Statistical details concerning compounds responsible for the clustering are given in tables S4 and S8.
(TIF)

Figure S4 **Effects of endophyte on aphid population growth, which was estimated by total aphid dry mass per plant.** Different letters over the bars indicate significant difference according to one-way ANOVA. ME-: manipulatively endophyte free; E-: naturally endophyte free; E+: naturally endophyte infected.
(TIF)

Figure S5 **Effects of endophyte by aphid on plant growth as estimated by aboveground dry weight.** Different letters over the bars indicate significant difference according to two-way ANOVA. NS: not significant. ME-: manipulatively endophyte free; E-: naturally endophyte free; E+: naturally endophyte infected. C: control; W: mechanical wounding; A: aphid infestation.
(TIF)

Table S1 **VOC emissions (ng gDW^−1^ h^−1^) from tall fescue at 12 days post feeding.** E-: naturally endophyte free; ME-: manipulatively endophyte free; E+: naturally endophyte infected.
(DOCX)

Table S2 **VOC emissions (ng gDW-1 h-1) from tall fescue at 6 days post feeding.** E-: naturally endophyte free; ME-: manipulatively endophyte free; E+: naturally endophyte infected.
(DOCX)

Table S3 **LMM results showing the endophyte effects in tall fescue after controlling for aphid damage.** In the model, endophyte, sampling time and their interaction were fixed factors, and aphid dry weight (an indicator of herbivory) was included as a covariate.
(DOCX)

Table S4 **VOC emissions (ng gDW-1 h-1) from tall fescue at 1 day after mechanical wounding.** E-: naturally endophyte free; ME-: manipulatively endophyte free; E+: naturally endophyte infected.
(DOCX)

Table S5 **VOC emissions (ng gDW-1 h-1) from meadow fescue at 12 days post feeding.** E-: naturally endophyte free; E+: naturally endophyte infected.
(DOCX)

Table S6 **VOC emissions (ng gDW-1 h-1) from meadow fescue at 6 days post damage.** E-: naturally endophyte free; E+: naturally endophyte infected.
(DOCX)

Table S7 **LMM results showing the endophyte effects in meadow fescue after controlling for aphid damage.** In the model, endophyte, sampling time and their interaction were fixed factors, and aphid dry weight (an indicator of herbivory) was included as a covariate.
(DOCX)

Table S8 **VOC emissions (ng gDW-1 h-1) from meadow fescue at 1 day after mechanical wounding.** E-: naturally endophyte free; E+: naturally endophyte infected.
(DOCX)
