# Supplementary figures and images for: Epichloë Endophytes Alter Inducible Indirect Defences in Host Grasses
Source: PLoS One. 2014 Jun 30;9(6):e101331. doi: 10.1371/journal.pone.0101331 (PMC4076332; doi:10.1371/journal.pone.0101331)

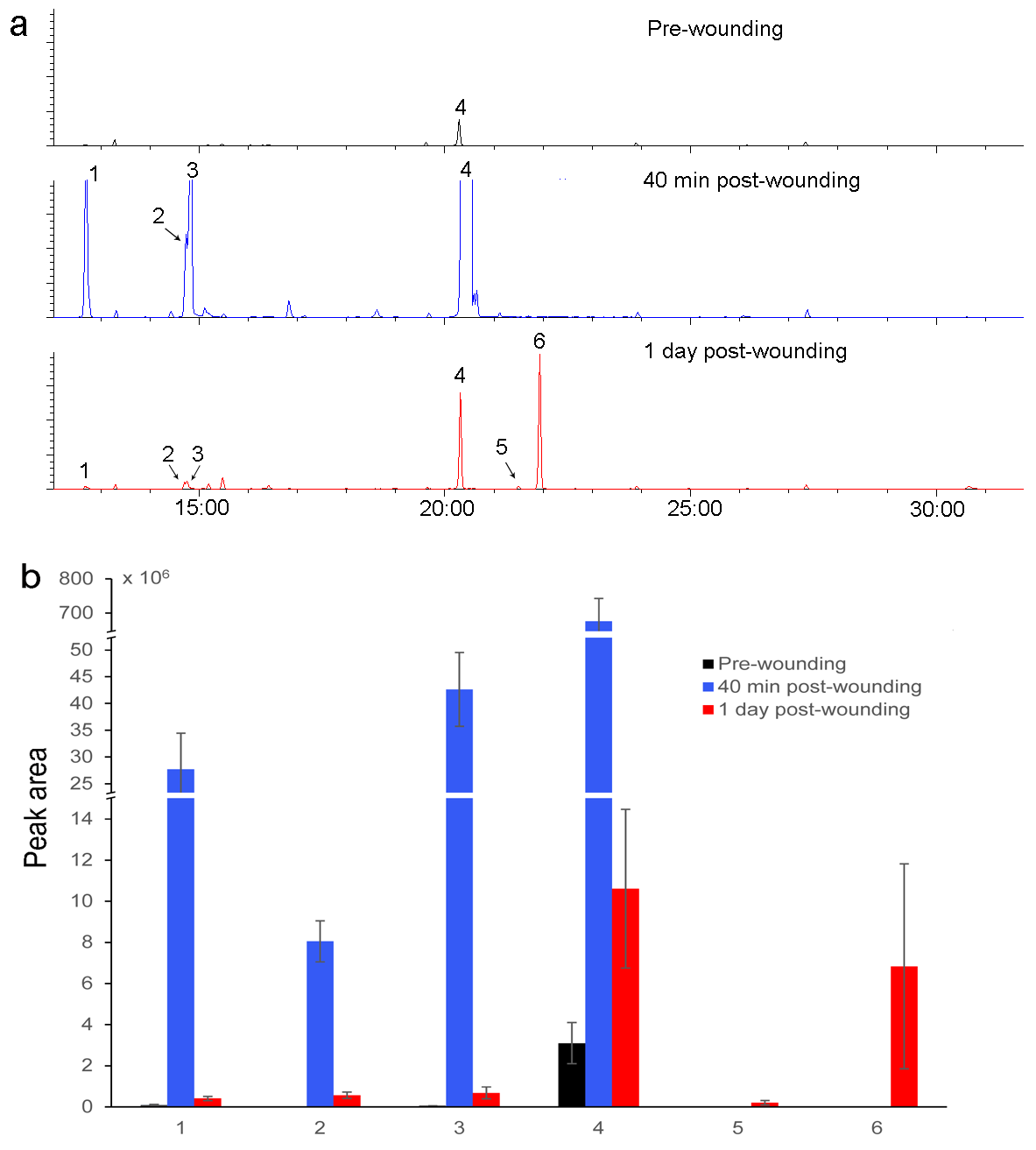

Supplement: Figure S1 — Kinetics of VOC emissions from red fescue ( Festuca rubra ) following mechanical wounding. (a) Total ion current (TIC) chromatograms of VOCs from a representative plant sample of red fescue before and after mechanical wounding. (b) Emissions (peak area ±SE; n = 4) of the dominant VOCs from red fescue. 1 = (Z)-3-hexenal, 2 = (E)-2-hexenal, 3 = (Z)-3-hexen-1-ol, 4 = (Z)-3-hexen-1-ol acetate, 5 = (Z)-β-ocimene, 6 = (E)-β-ocimene. (TIF) [file pone.0101331.s001.tif]

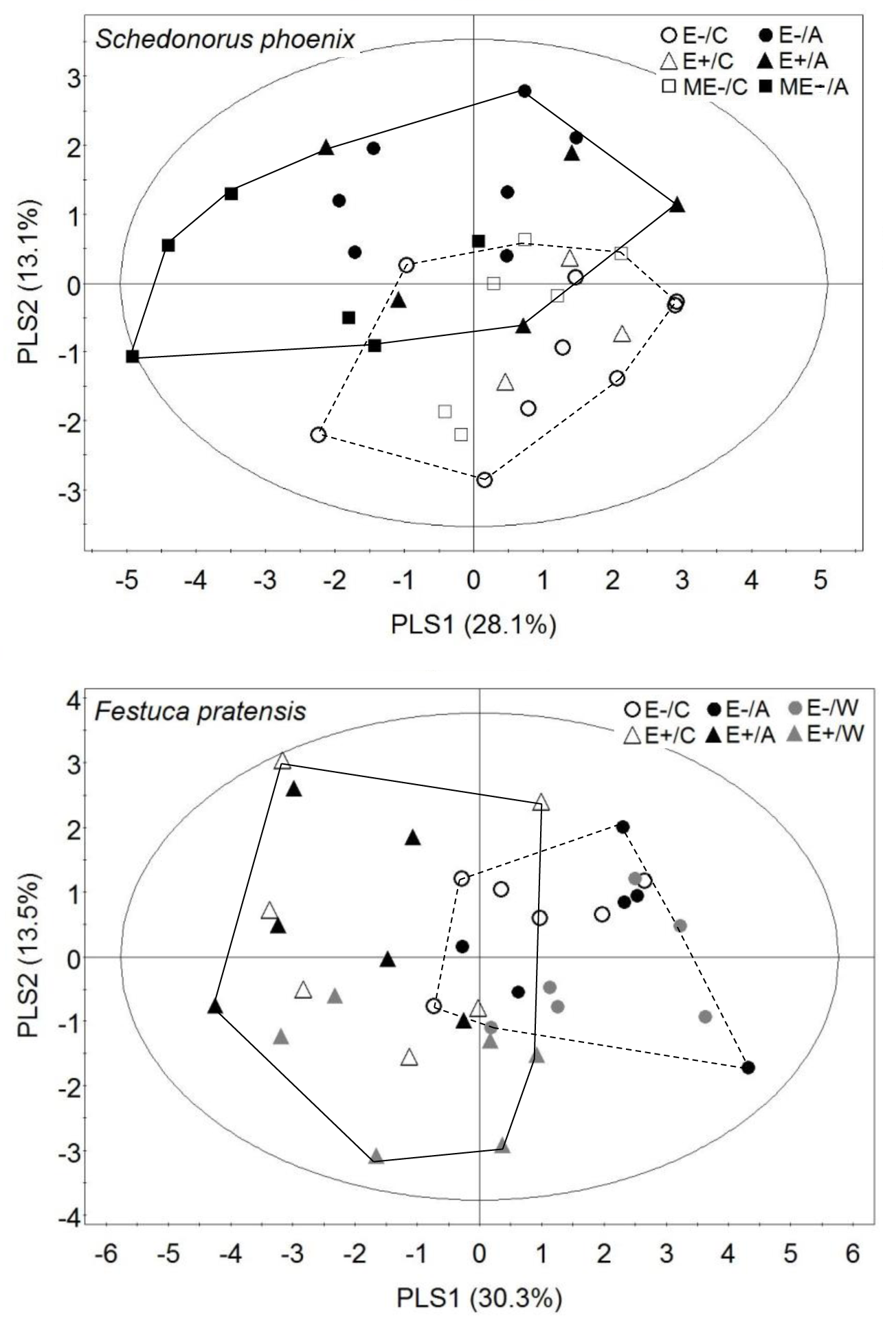

Supplement: Figure S2 — PLS-DA plots of VOC blends emitted by differently treated plants at 6 days after aphid addition. E-: naturally endophyte free; E+: naturally endophyte infected; ME-: manipulatively endophyte free; C: control; A: aphid feeding; W: mechanical wounding. For tall fescue (upper panel), a clear separation was seen between control and infested plants, whereas for meadow fescue (lower panel) the separation was mainly found between E- and E+ plants. Statistical details concerning compounds responsible for the clustering are given in table S2 and S6. (TIF) [file pone.0101331.s002.tif]

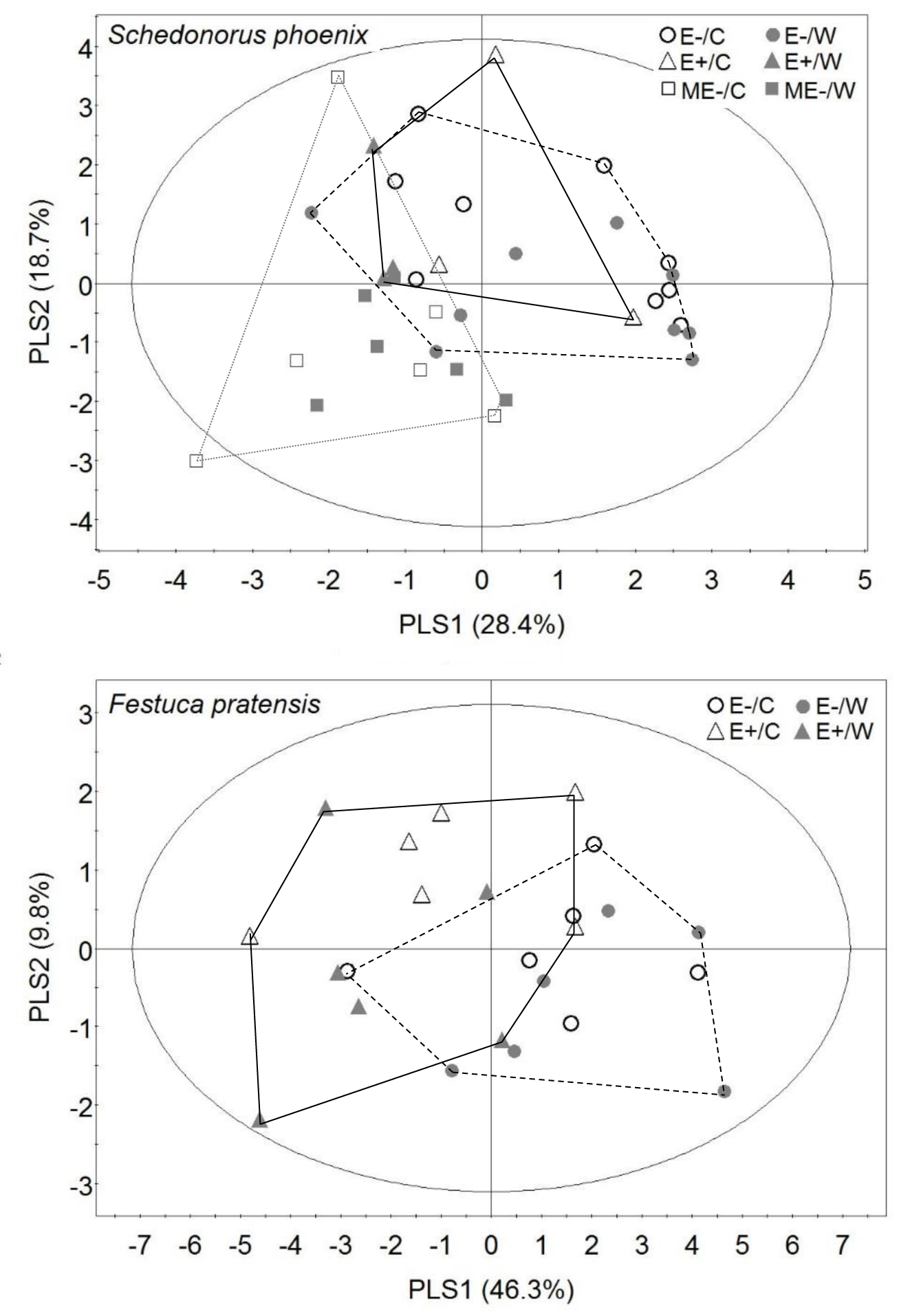

Supplement: Figure S3 — PLS-DA plots of VOC blends emitted by differently treated plants at 1 day after mechanical wounding. In tall fescue (upper panel) the strongest separation was observed between ME- plants and either of the E- and E+ plants, with the latter two largely overlapping. In meadow fescue (lower panel) E- and E+ plants, while overlapping somewhat, remained largely separated from each other (E-: naturally endophyte free; E+: naturally endophyte infected; ME-: manipulatively endophyte free; C: untreated control; W: mechanical wounding). Statistical details concerning compounds responsible for the clustering are given in tables S4 and S8. (TIF) [file pone.0101331.s003.tif]

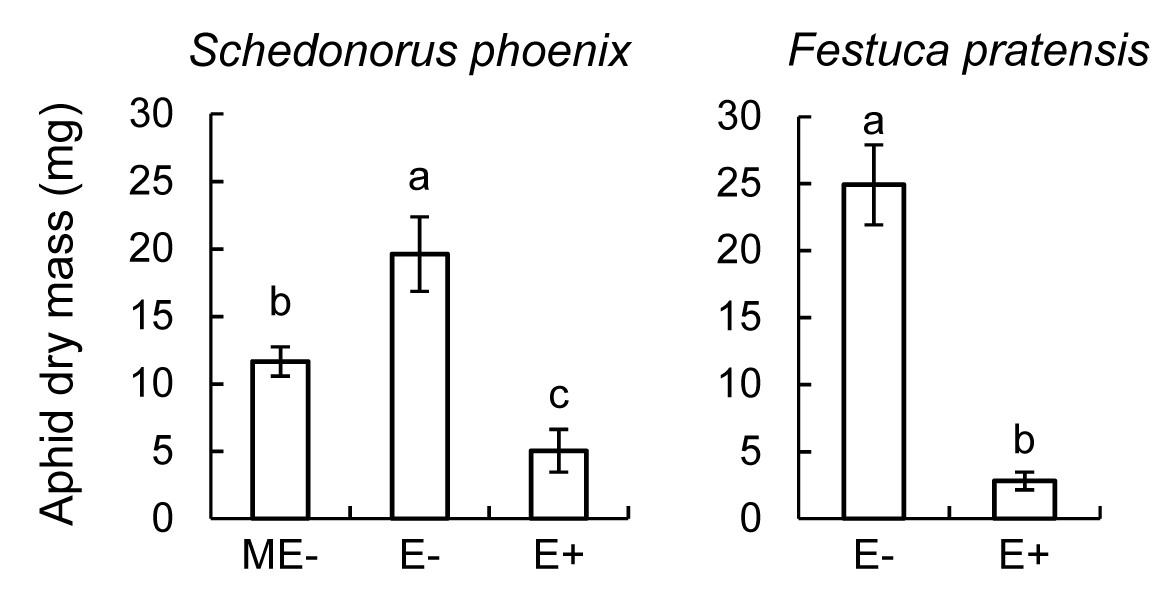

Supplement: Figure S4 — Effects of endophyte on aphid population growth, which was estimated by total aphid dry mass per plant. Different letters over the bars indicate significant difference according to one-way ANOVA. ME-: manipulatively endophyte free; E-: naturally endophyte free; E+: naturally endophyte infected. (TIF) [file pone.0101331.s004.tif]

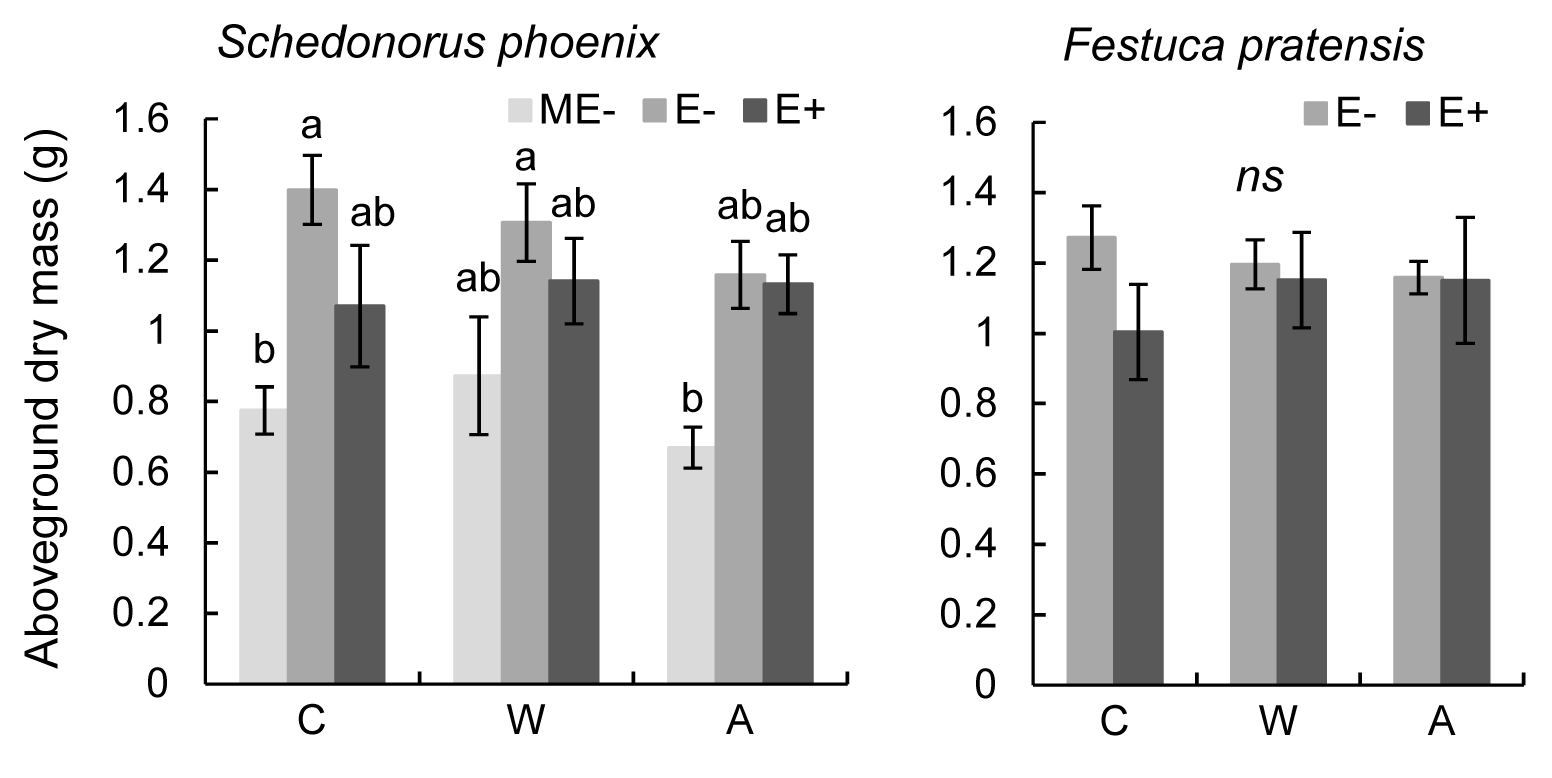

Supplement: Figure S5 — Effects of endophyte by aphid on plant growth as estimated by aboveground dry weight. Different letters over the bars indicate significant difference according to two-way ANOVA. NS: not significant. ME-: manipulatively endophyte free; E-: naturally endophyte free; E+: naturally endophyte infected. C: control; W: mechanical wounding; A: aphid infestation. (TIF) [file pone.0101331.s005.tif]
