# Supplementary material for: Epichloë Endophytes Alter Inducible Indirect Defences in Host Grasses
Source: PLoS One. 2014 Jun 30;9(6):e101331. doi: 10.1371/journal.pone.0101331 (PMC4076332; doi:10.1371/journal.pone.0101331)
Supplement: Table S1 — VOC emissions (ng gDW−1 h−1) from tall fescue at 12 days post feeding. E-: naturally endophyte free; ME-: manipulatively endophyte free; E+: naturally endophyte infected. (DOCX) [file pone.0101331.s006.docx]

Table S1. VOC emissions (ng gDW^-1^ h^-1^) from tall fescue at 12 days post feeding. E-: naturally endophyte free; ME-: manipulatively endophyte free; E+: naturally endophyte infected.

|  | Control | | | | | | | | |  | Aphid | | | | | | | | |  | *P*ǂ | | |  | VIP scores§ |
| --- | --- | --- | --- | --- | --- | --- | --- | --- | --- | --- | --- | --- | --- | --- | --- | --- | --- | --- | --- | --- | --- | --- | --- | --- | --- |
| Compound | E- (9) | | | ME- (6) | | | E+ (3) | | |  | E- (7) | | | ME- (6) | | | E+ (5) | | |  | E | A | ExA |  |  |
| Terpenoids |  |  |  |  |  |  |  |  |  |  |  |  |  |  |  |  |  |  |  |  |  |  |  |  |  |
| α-pinene | 0.29 | ± | 0.06 | 0.43 | ± | 0.16 | 0.26 | ± | 0.10 |  | 0.35 | ± | 0.10 | 0.60 | ± | 0.13 | 0.32 | ± | 0.11 |  | 0.165 | 0.332 | 0.815 |  | 0.65/0.71 |
| 6-methyl-5-hepten-2-one† | 0.19 | ± | 0.09 | 0.37 | ± | 0.34 | 0.23 | ± | 0.23 |  | 0.27 | ± | 0.10 | 0.34 | ± | 0.14 | 0.22 | ± | 0.12 |  | 0.859 | 0.670 | 0.969 |  | 0.35/0.35 |
| β-myrcene | 5.18 | ± | 0.84 | 5.04 | ± | 1.23 | 4.86 | ± | 1.45 |  | 5.02 | ± | 0.87 | 7.50 | ± | 1.31 | 4.19 | ± | 0.95 |  | 0.379 | 0.603 | 0.290 |  | 0.35/0.93 |
| β-pinene | 0.07 | ± | 0.05 | 0.25 | ± | 0.16 | 0.10 | ± | 0.10 |  | 0.17 | ± | 0.08 | 0.09 | ± | 0.09 | 0.16 | ± | 0.10 |  | 0.941 | 0.959 | 0.406 |  | 0.18/0.49 |
| δ-carene | 0.07 | ± | 0.05 | 0.17 | ± | 0.11 | 0.25 | ± | 0.25 |  | 0.30 | ± | 0.11 | 0.33 | ± | 0.21 | 0.27 | ± | 0.12 |  | 0.872 | 0.239 | 0.786 |  | 0.88/0.78 |
| (*Z)-*β-ocimene† | 5.48 | ± | 3.56 | 0.53 | ± | 0.25 | - |  |  |  | 3.62 | ± | 2.68 | 1.63 | ± | 0.41 | 0.93 | ± | 0.57 |  | 0.154 | 0.343 | 0.552 |  | 0.15/0.75 |
| d-limonene | 4.25 | ± | 0.69 | 3.63 | ± | 0.99 | 3.82 | ± | 1.23 |  | 3.65 | ± | 0.70 | 5.05 | ± | 0.75 | 3.12 | ± | 0.74 |  | 0.793 | 0.808 | 0.263 |  | 0.08/0.88 |
| β-phellandrene† | 0.22 | ± | 0.15 | 0.22 | ± | 0.22 | 0.30 | ± | 0.30 |  | 0.09 | ± | 0.09 | 0.21 | ± | 0.21 | - |  |  |  | 0.958 | 0.314 | 0.727 |  | 0.46/0.56 |
| (*E*)*-*β-ocimene | 3.26 | ± | 2.09 | - |  |  | - |  |  |  | 2.38 | ± | 1.79 | 0.86 | ± | 0.42 | 0.86 | ± | 0.56 |  | 0.203 | 0.331 | 0.553 |  | 0.12/0.56 |
| α-terpinolene | 0.84 | ± | 0.17 | 0.59 | ± | 0.28 | 0.74 | ± | 0.39 |  | 0.60 | ± | 0.23 | 0.75 | ± | 0.30 | 0.68 | ± | 0.21 |  | 0.920 | 0.835 | 0.643 |  | 0.31/0.56 |
| linalool | 1.05 | ± | 0.40 | 1.12 | ± | 0.23 | 1.45 | ± | 0.24 |  | 2.05 | ± | 0.28 | 2.49 | ± | 0.65 | 1.66 | ± | 0.39 |  | 0.750 | **0.022** | 0.412 |  | **1.54**/**1.35** |
| Unknown monoterpene† | 1.49 | ± | 0.20 | 1.99 | ± | 0.61 | 1.16 | ± | 0.58 |  | 1.37 | ± | 0.15 | 2.31 | ± | 0.55 | 1.61 | ± | 0.42 |  | 0.410 | 0.474 | 0.728 |  | 0.32/0.43 |
| (*E*)*-*β-caryophylene | 1.73 | ± | 0.53 | 0.87 | ± | 0.36 | 0.72 | ± | 0.72 |  | 3.13 | ± | 0.72 | 2.27 | ± | 0.64 | 1.43 | ± | 0.82 |  | **0.096** | **0.028** | 0.891 |  | **1.05**/**1.12** |
| Total Terpenoids | 23.95 | ± | 6.05 | 14.85 | ± | 3.33 | 13.67 | ± | 4.46 |  | 22.71 | ± | 6.08 | 24.09 | ± | 3.63 | 15.23 | ± | 3.15 |  | 0.304 | 0.274 | 0.420 |  |  |
| Green leaf volatiles (GLV) |  |  | |  |  | |  |  | |  |  |  | |  |  | |  |  | |  |  |  |  |  |  |
| (Z)-3-hexen-1-ol | 14.09 | ± | 4.37 | 58.71 | ± | 33.29 | 12.71 | ± | 1.30 |  | 36.90 | ± | 7.95 | 50.38 | ± | 30.16 | 57.61 | ± | 21.44 |  | 0.650 | **0.027** | 0.264 |  | **1.29**/**1.19** |
| (Z)-3-hexen-1-ol acetate | 412.71 | ± | 226.06 | 878.28 | ± | 352.66 | 157.06 | ± | 37.69 |  | 439.69 | ± | 93.65 | 696.75 | ± | 445.41 | 1150.02 | ± | 445.34 |  | 0.605 | 0.109 | 0.317 |  | 0.90/0.83 |
| Total GLV | 426.80 | ± | 230.34 | 936.99 | ± | 381.25 | 169.77 | ± | 36.86 |  | 476.59 | ± | 99.12 | 747.13 | ± | 475.44 | 1207.64 | ± | 461.08 |  | 0.619 | **0.049** | 0.309 |  |  |
| Other compounds |  |  | |  |  | |  |  | |  |  |  | |  |  | |  |  | |  |  |  |  |  |  |
| 1-octen-3-ol | - |  |  | 0.52 | ± | 0.52 | - |  |  |  | 47.96 | ± | 5.95 | 47.55 | ± | 7.92 | 37.48 | ± | 11.03 |  | 0.343 | <**0.000** | 0.537 |  | **3.02**/**2.64** |
| methyl salicylate | 2.13 | ± | 1.06 | 2.31 | ± | 0.94 | 1.32 | ± | 0.98 |  | 0.73 | ± | 0.29 | 2.59 | ± | 0.55 | 3.15 | ± | 2.25 |  | 0.235 | 0.809 | 0.330 |  | 0.01/0.23 |
| Total VOCs | 453.08 | ± | 228.16 | 955.04 | ± | 381.45 | 184.99 | ± | 41.77 |  | 548.28 | ± | 97.92 | 821.70 | ± | 483.60 | 1263.71 | ± | 468.34 |  | 0.712 | **0.053** | 0.294 |  |  |

ǂ Bold numbers indicate significant or marginally significant effects of endophyte (E), aphid (A) or their interaction (E×A) as determined by individual two-way ANOVAs based on log-transformed data. Numbers within the brackets denote sample size.

§ Variable Importance in the Projection (VIP) scores for PLS-DA are given for the first three components, which are separated by slashes. VIP scores highlighted in bold are higher than 1 and are most influential for separation of individual treatments.

† Compounds are tentatively identified.
