# Supplementary material for: Epichloë Endophytes Alter Inducible Indirect Defences in Host Grasses
Source: PLoS One. 2014 Jun 30;9(6):e101331. doi: 10.1371/journal.pone.0101331 (PMC4076332; doi:10.1371/journal.pone.0101331)
Supplement: Table S2 — VOC emissions (ng gDW-1 h-1) from tall fescue at 6 days post feeding. E-: naturally endophyte free; ME-: manipulatively endophyte free; E+: naturally endophyte infected. (DOCX) [file pone.0101331.s007.docx]

Table S2. VOC emissions (ng gDW^-1^ h^-1^) from tall fescue at 6 days post feeding. E-: naturally endophyte free; ME-: manipulatively endophyte free; E+: naturally endophyte infected.

|  | Control | | | | | | | | |  | Aphid | | | | | | | | |  | *P*ǂ | | |  | VIP scores§ |
| --- | --- | --- | --- | --- | --- | --- | --- | --- | --- | --- | --- | --- | --- | --- | --- | --- | --- | --- | --- | --- | --- | --- | --- | --- | --- |
| Compound | E- (9) | | | ME- (6) | | | E+ (3) | | |  | E- (7) | | | ME- (6) | | | E+ (5) | | |  | E | A | E×A |  |  |
| Terpenoids |  |  |  |  |  |  |  |  |  |  |  |  |  |  |  |  |  |  |  |  |  |  |  |  |  |
| α-pinene | 0.87 | ± | 0.06 | 1.39 | ± | 0.22 | 0.96 | ± | 0.13 |  | 0.88 | ± | 0.10 | 1.67 | ± | 0.18 | 0.94 | ± | 0.09 |  | **0.000** | 0.487 | 0.510 |  | **1.60**/**1.31**/**1.61** |
| 6-methyl-5-hepten-2-one† | 0.24 | ± | 0.13 | 0.34 | ± | 0.16 | 0.10 | ± | 0.10 |  | 0.34 | ± | 0.16 | 0.55 | ± | 0.38 | 0.44 | ± | 0.28 |  | 0.795 | 0.350 | 0.881 |  | 0.48/0.33/0.26 |
| β-myrcene | 8.64 | ± | 1.97 | 4.59 | ± | 1.53 | 6.42 | ± | 1.91 |  | 6.89 | ± | 1.37 | 12.67 | ± | 3.05 | 5.23 | ± | 1.43 |  | 0.697 | 0.339 | **0.033** |  | **1.01**/0.93/**1.14** |
| β-pinene | 0.06 | ± | 0.02 | 0.08 | ± | 0.03 | 0.03 | ± | 0.03 |  | 0.04 | ± | 0.02 | 0.08 | ± | 0.05 | 0.04 | ± | 0.03 |  | 0.327 | 0.973 | 0.786 |  | 0,35/0,52/0,65 |
| δ-carene | 0.18 | ± | 0.04 | 0.26 | ± | 0.08 | 0.30 | ± | 0.09 |  | 0.26 | ± | 0.06 | 0.49 | ± | 0.15 | 0.29 | ± | 0.10 |  | 0.251 | 0.253 | 0.503 |  | **1.21**/0.83/0.86 |
| (*Z)-*β-ocimene† | 3.42 | ± | 2.76 | 0.20 | ± | 0.20 | - |  |  |  | 2.12 | ± | 1.02 | 2.21 | ± | 0.64 | 1.28 | ± | 0.68 |  | 0.379 | **0.042** | 0.327 |  | 0.88/0.62/0.93 |
| d-limonene | 5.39 | ± | 1.49 | 3.02 | ± | 1.33 | 3.30 | ± | 1.82 |  | 3.27 | ± | 0.94 | 5.48 | ± | 1.72 | 2.40 | ± | 1.03 |  | 0.607 | 0.886 | 0.228 |  | 0.38/**1.05**/**1.22** |
| β-phellandrene† | 0.68 | ± | 0.31 | 0.55 | ± | 0.35 | 0.47 | ± | 0.47 |  | 0.43 | ± | 0.30 | 1.03 | ± | 0.56 | 0.47 | ± | 0.20 |  | 0.842 | 0.823 | 0.652 |  | 0.38/0.72/0.66 |
| (*E*)*-*β-ocimene | 1.76 | ± | 1.65 | 0.69 | ± | 0.31 | - |  |  |  | 1.74 | ± | 0.81 | 1.48 | ± | 0.78 | 0.96 | ± | 0.52 |  | 0.641 | 0.151 | 0.888 |  | 0.60/0.60/0.50 |
| α-terpinolene | 1.39 | ± | 0.36 | 1.04 | ± | 0.45 | 0.84 | ± | 0.50 |  | 0.98 | ± | 0.30 | 1.46 | ± | 0.54 | 0.76 | ± | 0.31 |  | 0.692 | 0.922 | 0.670 |  | 0.12/0.83/0.96 |
| linalool | 1.27 | ± | 0.39 | 1.53 | ± | 0.40 | 1.74 | ± | 0.52 |  | 2.28 | ± | 0.38 | 4.12 | ± | 1.32 | 2.39 | ± | 1.00 |  | 0.403 | **0.039** | 0.416 |  | **1.53**/**1.09**/**0.87** |
| Unknown monoterpene† | 2.15 | ± | 0.38 | 2.86 | ± | 0.98 | 1.72 | ± | 0.31 |  | 2.01 | ± | 0.46 | 4.95 | ± | 1.14 | 1.57 | ± | 0.37 |  | **0.013** | 0.447 | 0.183 |  | **1.51**/**1.38**/**1.13** |
| (*E*)*-*β-caryophylene | 2.82 | ± | 0.83 | 2.07 | ± | 0.71 | 0.59 | ± | 0.59 |  | 4.36 | ± | 1.25 | 2.95 | ± | 0.89 | 1.20 | ± | 0.77 |  | **0.047** | 0.236 | 0.954 |  | 0.71/0.51/0.85 |
| Total Terpenoids | 28.63 | ± | 6.68 | 18.27 | ± | 3.87 | 16.37 | ± | 5.47 |  | 25.25 | ± | 4.31 | 38.59 | ± | 8.71 | 17.53 | ± | 5.31 |  | 0.208 | 0.321 | 0.302 |  |  |
| Green leaf volatiles (GLV) |  |  | |  |  | |  |  | |  |  |  | |  |  | |  |  | |  |  |  |  |  |  |
| (Z)-3-hexen-1-ol | 18.42 | ± | 5.45 | 62.60 | ± | 22.91 | 33.95 | ± | 14.68 |  | 33.08 | ± | 4.53 | 29.29 | ± | 11.72 | 29.86 | ± | 11.37 |  | 0.721 | 0.985 | 0.147 |  | 0.09/0.45/0.67 |
| (Z)-3-hexen-1-ol acetate | 152.59 | ± | 52.24 | 658.92 | ± | 298.97 | 301.31 | ± | 146.42 |  | 446.69 | ± | 153.03 | 238.95 | ± | 89.53 | 358.55 | ± | 151.45 |  | 0.752 | 0.793 | 0.181 |  | 0.04/0.27/0.55 |
| Total GLV | 171.01 | ± | 57.41 | 721.52 | ± | 318.67 | 335.27 | ± | 156.51 |  | 479.77 | ± | 155.32 | 268.25 | ± | 99.15 | 388.41 | ± | 162.13 |  | 0.759 | 0.986 | 0.134 |  |  |
| Other compounds |  |  | |  |  | |  |  | |  |  |  | |  |  | |  |  | |  |  |  |  |  |  |
| 1-octen-3-ol | - |  |  | - |  |  | - |  |  |  | 10.66 | ± | 2.29 | 7.41 | ± | 3.73 | 5.90 | ± | 2.76 |  | 0.229 | **0.000** | 0.229 |  | **1.39**/**2.27**/**1.89** |
| methyl salicylate | 1.28 | ± | 0.84 | 1.26 | ± | 0.94 | 0.51 | ± | 0.51 |  | 2.87 | ± | 2.11 | 6.78 | ± | 2.33 | 2.31 | ± | 0.91 |  | 0.173 | **0.007** | 0.243 |  | **1.75**/**1.20**/0.97 |
| Total VOCs | 201.16 | ± | 55.34 | 741.38 | ± | 321.25 | 352.24 | ± | 161.16 |  | 518.90 | ± | 156.87 | 321.57 | ± | 104.34 | 414.60 | ± | 167.50 |  | 0.705 | 0.994 | 0.206 |  |  |

ǂ Bold numbers indicate significant or marginally significant effects of endophyte (E), aphid (A) or their interaction (E×A) as determined by individual two-way ANOVAs based on log-transformed data. Numbers within the brackets denote sample size.

§ Variable Importance in the Projection (VIP) scores for PLS-DA are given for the first three components, which are separated by slashes. VIP scores highlighted in bold are higher than 1 and are most influential for separation of individual treatments.

† Compounds are tentatively identified.
