# Supplementary material for: Epichloë Endophytes Alter Inducible Indirect Defences in Host Grasses
Source: PLoS One. 2014 Jun 30;9(6):e101331. doi: 10.1371/journal.pone.0101331 (PMC4076332; doi:10.1371/journal.pone.0101331)
Supplement: Table S3 — LMM results showing the endophyte effects in tall fescue after controlling for aphid damage. In the model, endophyte, sampling time and their interaction were fixed factors, and aphid dry weight (an indicator of herbivory) was included as a covariate. (DOCX) [file pone.0101331.s008.docx]

Table S3. LMM results showing the endophyte effects in tall fescue after controlling for aphid damage. In the model, endophyte, sampling time and their interaction were fixed factors, and aphid dry weight (an indicator of herbivory) was included as a covariate.

| Compound | Endophyte | |  | Sampling date | |  | Interaction | |  | Aphid dry weight | |  | Day 6 | Day 12 |
| --- | --- | --- | --- | --- | --- | --- | --- | --- | --- | --- | --- | --- | --- | --- |
|  | *F* | *P* |  | *F* | *P* |  | *F* | *P* |  | *F* | *P* |  | *P*ǂ | *P*ǂ |
| Terpenoids |  |  |  |  |  |  |  |  |  |  |  |  |  |  |
| α-pinene | *F*_2, 27.7_ = 8.03 | **0,002** |  | *F*_1, 26.2_ = 48.8 | < **0.001** |  | *F*_2, 26.2_ = 0.82 | 0,448 |  | *F*_1, 25.8_ = 0.01 | 0,941 |  | **0,002** | 0,247 |
| 6-methyl-5-hepten-2-one† | *F*_2, 24.6_ = 0.43 | 0,653 |  | *F*_1, 21.0_ = 0.33 | 0,571 |  | *F*_2, 21.0_ = 0.04 | 0,952 |  | *F*_1, 20.2_ = 1.09 | 0,307 |  | 0,770 | 0,686 |
| β-myrcene | *F*_2, 28.2_ = 5.51 | **0,009** |  | *F*_1, 27.1_ = 2.58 | 0,119 |  | *F*_2, 27.1_ = 0.20 | 0,813 |  | *F*_1, 26.8_ = 0.04 | 0,836 |  | **0,070** | 0,112 |
| β-pinene | *F*_2, 23.3_ = 0.25 | 0,776 |  | *F*_1, 19.5_ = 2.09 | 0,164 |  | *F*_2, 19.5_ = 0.62 | 0,547 |  | *F*_1, 18.6_ = 1.21 | 0,284 |  | 0,361 | 0,749 |
| δ-carene | *F*_2, 26.0_ = 1.17 | 0,326 |  | *F*_1, 23.2_ = 0.35 | 0,558 |  | *F*_2, 23.2_ = 0.40 | 0,673 |  | *F*_1, 22.6_ = 3.84 | **0,062** |  | 0,185 | 0,556 |
| (*Z*)-β-ocimene† | *F*_2, 28.8_ = 0.83 | 0,442 |  | *F*_1, 28.6_ = 0.11 | 0,734 |  | *F*_2, 28.6_ = 0.12 | 0,880 |  | *F*_1, 28.6_ = 0.25 | 0,615 |  | 0,652 | 0,543 |
| d-limonene | *F*_2, 25.3_ = 2.52 | 0,100 |  | *F*_1, 22.1_ = 1.22 | 0,280 |  | *F*_2, 22.1_ = 0.16 | 0,853 |  | *F*_1, 21.4_ < 0.01 | 0,973 |  | 0,317 | 0,195 |
| β-phellandrene† | *F*_2, 23.8_ = 1.13 | 0,337 |  | *F*_1, 20.1_ = 5.71 | **0,027** |  | *F*_2, 20.1_ = 0.26 | 0,768 |  | *F*_1, 29.2_ = 1.08 | 0,312 |  | 0,469 | 0,555 |
| (*E*)*-*β-ocimene | *F*_2, 28.9_ = 0.19 | 0,825 |  | *F*_1, 28.8_ = 0.17 | 0,675 |  | *F*_2, 28.8_ = 0.01 | 0,990 |  | *F*_1, 28.8_ = 0.05 | 0,825 |  | 0,879 | 0,860 |
| α-terpinolene | *F*_2, 28.1_ = 0.33 | 0,717 |  | *F*_1, 27.0_ = 1.06 | 0,312 |  | *F*_2, 27.0_ = 0.23 | 0,794 |  | *F*_1, 26.8_ = 0.11 | 0,739 |  | 0,713 | 0,869 |
| linalool | *F*_2, 26.8_ = 1.97 | 0,158 |  | *F*_1, 24.6_ = 1.09 | 0,306 |  | *F*_2, 24.6_ = 0.40 | 0,672 |  | *F*_1, 24.1_ = 0.19 | 0,660 |  | 0,267 | 0,576 |
| Unknown monoterpene† | *F*_2, 29.0_ = 5.34 | **0,011** |  | *F*_1, 28.9_ = 3.73 | **0,063** |  | *F*_2, 28.9_ = 1.72 | 0,197 |  | *F*_1, 28.9_ = 0.05 | 0,827 |  | **0,008** | 0,595 |
| (*E*)*-*β*-*caryophylene | *F*_2, 28.3_ = 2.27 | 0,121 |  | *F*_1, 27.4_ = 0.07 | 0,794 |  | *F*_2, 27.4_ = 0.22 | 0,804 |  | *F*_1, 27.2_ < 0.01 | 0,940 |  | 0,179 | 0,360 |
| Total Terpenoids | *F*_2, 28.5_ = 3.49 | **0,044** |  | *F*_1, 27.8_ = 0.96 | 0,333 |  | *F*_2, 27.8_ = 0.31 | 0,730 |  | *F*_1, 27.7_ < 0.01 | 0,990 |  | 0,110 | 0,342 |
| Green leaf volatiles (GLV) |  |  |  |  |  |  |  |  |  |  |  |  |  |  |
| (*Z*)-3-hexen-1-ol | *F*_2, 28..8_ = 0.63 | 0,536 |  | *F*_1, 28.5_ = 1.50 | 0,230 |  | *F*_2, 28.5_ = 0.59 | 0,557 |  | *F*_1, 28.5_ = 0.30 | 0,586 |  | 0,518 | 0,674 |
| (*Z*)-3-hexen-1-ol acetate | *F*_2, 28.3_ = 0.37 | 0,689 |  | *F*_1, 27.5_ = 4.55 | **0,042** |  | *F*_2, 27.5_ = 0.65 | 0,532 |  | *F*_1, 27.3_ = 0.38 | 0,543 |  | 0,630 | 0,721 |
| Total GLV | *F*_2, 28.6_ = 0.43 | 0,649 |  | *F*_1, 28.0_ = 4.30 | **0,047** |  | *F*_2, 28.0_ = 0.71 | 0,499 |  | *F*_1, 27.8_ = 0.40 | 0,530 |  | 0,587 | 0,704 |
| Other compounds |  |  |  |  |  |  |  |  |  |  |  |  |  |  |
| 1-octen-3-ol | *F*_2, 22.7_ = 0.26 | 0,772 |  | *F*_1, 18.9_ = 50.6 | < **0.001** |  | *F*_2, 18.9_ = 1.06 | 0,364 |  | *F*_1, 18.0_ = 2.22 | 0,153 |  | 0,518 | 0,707 |
| methyl salicylate | *F*_2, 27.8_ = 4.36 | **0,022** |  | *F*_1, 26.3_ = 1.85 | 0,185 |  | *F*_2, 26.3_ = 0.48 | 0,619 |  | *F*_1, 26.0_ = 0.02 | 0,871 |  | **0,086** | 0,164 |
| Total VOCs | *F*_2, 28.6_ = 0.31 | 0,734 |  | *F*_1, 28.2_ = 4.84 | **0,036** |  | *F*_2, 28.2_ = 0.72 | 0,492 |  | *F*_1, 28.6_ = 0.33 | 0,569 |  | 0,621 | 0,734 |

ǂ Sidak-adjusted pairwise comparisons were made to compare the endophyte effects within each sampling time point. Bold numbers indicate significant or marginally significant effects.

† Compounds are tentatively identified.
