# Supplementary material for: Epichloë Endophytes Alter Inducible Indirect Defences in Host Grasses
Source: PLoS One. 2014 Jun 30;9(6):e101331. doi: 10.1371/journal.pone.0101331 (PMC4076332; doi:10.1371/journal.pone.0101331)
Supplement: Table S4 — VOC emissions (ng gDW-1 h-1) from tall fescue at 1 day after mechanical wounding. E-: naturally endophyte free; ME-: manipulatively endophyte free; E+: naturally endophyte infected. (DOCX) [file pone.0101331.s009.docx]

Table S4. VOC emissions (ng gDW^-1^ h^-1^) from tall fescue at 1 day after mechanical wounding. E-: naturally endophyte free; ME-: manipulatively endophyte free; E+: naturally endophyte infected.

|  | Control | | | | | | | | |  | Wounding | | | | | | | | |  | *P*ǂ | | |  | VIP scores§ |
| --- | --- | --- | --- | --- | --- | --- | --- | --- | --- | --- | --- | --- | --- | --- | --- | --- | --- | --- | --- | --- | --- | --- | --- | --- | --- |
| Compound | E- (9) | | | ME- (6) | | | E+ (3) | | |  | E- (9) | | | ME- (6) | | | E+ (3) | | |  | E | W | E×W |  |  |
| Terpenoids |  |  |  |  |  |  |  |  |  |  |  |  |  |  |  |  |  |  |  |  |  |  |  |  |  |
| α-pinene | 0.91 | ± | 0.48 | 3.10 | ± | 1.41 | 1.17 | ± | 1.17 |  | 1.13 | ± | 0.68 | 1.85 | ± | 0.86 | 3.19 | ± | 0.66 |  | 0.202 | 0.407 | 0.343 |  | **1.26/1.05** |
| 6-methyl-5-hepten-2-one† | 0.68 | ± | 0.47 | 0.84 | ± | 0.62 | 0.84 | ± | 0.43 |  | 0.09 | ± | 0.06 | 1.28 | ± | 0.85 | 0.23 | ± | 0.15 |  | 0.259 | 0.400 | 0.403 |  | 0.77/0.61 |
| β-myrcene | 8.06 | ± | 2.59 | 5.81 | ± | 3.43 | 9.64 | ± | 6.78 |  | 5.10 | ± | 1.83 | 5.66 | ± | 1.24 | 7.45 | ± | 4.41 |  | 0.929 | 0.971 | 0.609 |  | 0.15/0.82 |
| β-pinene | 0.13 | ± | 0.07 | 0.47 | ± | 0.18 | 0.08 | ± | 0.08 |  | 0.15 | ± | 0.09 | 0.30 | ± | 0.10 | 0.39 | ± | 0.15 |  | **0.093** | 0.514 | 0.280 |  | **1.54/1.19** |
| δ-carene | 0.41 | ± | 0.16 | 1.50 | ± | 0.43 | 0.53 | ± | 0.27 |  | 0.46 | ± | 0.21 | 1.23 | ± | 0.13 | 0.73 | ± | 0.30 |  | **0.001** | 0.816 | 0.869 |  | **2.04/1.64** |
| (*Z*)-β-ocimene† | 1.05 | ± | 0.73 | 2.25 | ± | 0.83 | - |  |  |  | 1.06 | ± | 0.50 | 2.38 | ± | 1.42 | 0.46 | ± | 0.46 |  | 0.126 | 0.751 | 0.808 |  | 0.84/0.76 |
| d-limonene | 6.11 | ± | 2.22 | 6.83 | ± | 2.34 | 8.07 | ± | 4.78 |  | 4.11 | ± | 1.71 | 4.37 | ± | 1.46 | 8.10 | ± | 2.80 |  | 0.433 | 0.811 | 0.701 |  | **0.73/1.09** |
| β-phellandrene† | 0.40 | ± | 0.26 | 0.44 | ± | 0.44 | 0.79 | ± | 0.79 |  | 0.18 | ± | 0.18 | - |  |  | 0.47 | ± | 0.47 |  | 0.521 | 0.351 | 0.948 |  | 0.09/0.98 |
| (*E*)*-*β-ocimene | 0.62 | ± | 0.42 | 1.65 | ± | 0.61 | 0.24 | ± | 0.24 |  | 0.64 | ± | 0.34 | 1.69 | ± | 0.94 | 0.63 | ± | 0.31 |  | 0.162 | 0.775 | 0.819 |  | **1.11/0.88** |
| α-terpinolene | 1.40 | ± | 0.51 | 0.71 | ± | 0.71 | 1.59 | ± | 1.09 |  | 0.69 | ± | 0.35 | 0.44 | ± | 0.28 | 1.39 | ± | 0.80 |  | 0.283 | 0.647 | 0.792 |  | **0.53/1.40** |
| linalool | 1.12 | ± | 0.48 | 2.91 | ± | 0.65 | 2.02 | ± | 1.08 |  | 1.55 | ± | 0.78 | 3.21 | ± | 0.64 | 2.97 | ± | 0.15 |  | **0.015** | 0.364 | 0.823 |  | **1.75/1.35** |
| Unknown monoterpene† | 2.67 | ± | 0.45 | 3.30 | ± | 1.42 | 3.34 | ± | 1.38 |  | 1.41 | ± | 0.31 | 1.74 | ± | 0.24 | 2.79 | ± | 0.83 |  | 0.344 | 0.128 | 0.714 |  | **0.56/1.16** |
| (*E*)*-*β*-*caryophylene | 2.86 | ± | 1.35 | 1.71 | ± | 0.55 | 0.99 | ± | 0.99 |  | 3.45 | ± | 1.17 | 2.11 | ± | 0.70 | 2.14 | ± | 1.47 |  | 0.571 | 0.414 | 0.897 |  | 0.32/0.25 |
| Total Terpenoids | 25.74 | ± | 8.23 | 30.67 | ± | 9.13 | 28.46 | ± | 16.47 |  | 19.93 | ± | 7.20 | 24.98 | ± | 4.94 | 30.71 | ± | 10.81 |  | 0.228 | 0.855 | 0.763 |  |  |
| Green leaf volatiles (GLV) |  |  | |  |  | |  |  | |  |  |  | |  |  | |  |  | |  |  |  |  |  |  |
| (*Z*)-3-hexen-1-ol | 12.43 | ± | 3.90 | 16.52 | ± | 6.04 | 7.03 | ± | 3.33 |  | 15.63 | ± | 4.33 | 15.79 | ± | 7.05 | 4.43 | ± | 2.60 |  | 0.296 | 0.826 | 0.553 |  | 0.47/0.47 |
| (*Z*)-3-hexen-1-ol acetate | 33.35 | ± | 13.07 | 33.23 | ± | 14.91 | 13.25 | ± | 6.42 |  | 39.08 | ± | 19.71 | 66.87 | ± | 47.32 | 11.11 | ± | 2.28 |  | 0.646 | 0.892 | 0.806 |  | 0.07/0.10 |
| Total GLV | 45.79 | ± | 16.20 | 49.75 | ± | 20.36 | 20.29 | ± | 7.81 |  | 54.71 | ± | 23.95 | 82.66 | ± | 54.08 | 15.54 | ± | 3.32 |  | 0.626 | 0.965 | 0.738 |  |  |
| Other compounds |  |  | |  |  | |  |  | |  |  |  | |  |  | |  |  | |  |  |  |  |  |  |
| 1-octen-3-ol | - |  |  | - |  |  | - |  |  |  | - |  |  | - |  |  | - |  |  |  |  |  |  |  |  |
| methyl salicylate | - |  |  | 0.12 | ± | 0.12 | - |  |  |  | - |  |  | - |  |  | 1.12 | ± | 1.12 |  | 0.104 | 0.135 | 0.054 |  | 0.91/0.84 |
| Total VOCs | 72.21 | ± | 24.19 | 81.38 | ± | 27.43 | 49.58 | ± | 10.25 |  | 74.73 | ± | 30.43 | 108.92 | ± | 57.04 | 47.60 | ± | 9.52 |  | 0.587 | 0.857 | 0.950 |  |  |

ǂ Bold numbers indicate significant or marginally significant effects of endophyte (E), wounding (W) or their interaction (E×W) as determined by individual two-way ANOVAs based on log-transformed data. Numbers within the brackets denote sample size.

§ Variable Importance in the Projection (VIP) scores for PLS-DA are given for the first three components, which are separated by slashes. VIP scores highlighted in bold are higher than 1 and are most influential for separation of individual treatments.

† Compounds are tentatively identified.
