# Supplementary material for: Epichloë Endophytes Alter Inducible Indirect Defences in Host Grasses
Source: PLoS One. 2014 Jun 30;9(6):e101331. doi: 10.1371/journal.pone.0101331 (PMC4076332; doi:10.1371/journal.pone.0101331)
Supplement: Table S5 — VOC emissions (ng gDW-1 h-1) from meadow fescue at 12 days post feeding. E-: naturally endophyte free; E+: naturally endophyte infected. (DOCX) [file pone.0101331.s010.docx]

Table S5. VOC emissions (ng gDW^-1^ h^-1^) from meadow fescue at 12 days post feeding. E-: naturally endophyte free; E+: naturally endophyte infected.

|  | Control | | | | | |  | Aphid | | | | | |  | *P*ǂ | | |  |  |
| --- | --- | --- | --- | --- | --- | --- | --- | --- | --- | --- | --- | --- | --- | --- | --- | --- | --- | --- | --- |
| Compound | E- (6) | | | E+ (6) | | |  | E- (6) | | | E+ (6) | | |  | E | A | E**×**A |  | VIP scores§ |
| Terpenoids |  |  |  |  |  |  |  |  |  |  |  |  |  |  |  |  |  |  |  |
| α-pinene | 1.75 | ± | 0.19 | 2.09 | ± | 0.24 |  | 2.40 | ± | 0.33 | 1.98 | ± | 0.35 |  | 0.890 | 0.439 | 0.183 |  | 0.62/0.56/0.78 |
| 6-methyl-5-hepten-2-one† | 0.41 | ± | 0.18 | 0.44 | ± | 0.28 |  | 0.93 | ± | 0.22 | 0.04 | ± | 0.04 |  | **0.017** | 0.754 | **0.026** |  | **1.37**/**1.10**/**1.18** |
| β-myrcene | 14.02 | ± | 3.35 | 5.28 | ± | 1.86 |  | 21.37 | ± | 4.47 | 8.18 | ± | 2.36 |  | **0.004** | 0.216 | 0.912 |  | **1.32**/**1.14**/**1.17** |
| β-pinene | 0.38 | ± | 0.12 | 0.55 | ± | 0.13 |  | 0.63 | ± | 0.15 | 0.53 | ± | 0.13 |  | 0.768 | 0.432 | 0.353 |  | 0.35/0.52/0.65 |
| δ-carene | 1.27 | ± | 0.10 | 1.40 | ± | 0.32 |  | 1.54 | ± | 0.20 | 1.45 | ± | 0.28 |  | 0.794 | 0.495 | 0.832 |  | 0.37/0.34/0.30 |
| (*Z*)-β-ocimene† | 0.42 | ± | 0.27 | 1.80 | ± | 0.70 |  | 12.28 | ± | 8.59 | 2.46 | ± | 0.64 |  | 0.899 | **0.017** | **0.086** |  | **1.07**/**1.22**/**1.10** |
| d-limonene | 10.36 | ± | 2.09 | 5.25 | ± | 1.37 |  | 14.63 | ± | 2.96 | 9.62 | ± | 3.04 |  | **0.015** | **0.070** | 0.643 |  | **1.23**/0.95/**1.18** |
| β-phellandrene† | 1.83 | ± | 0.32 | 0.79 | ± | 0.30 |  | 2.04 | ± | 0.66 | 1.10 | ± | 0.36 |  | **0.037** | 0.707 | 0.615 |  | 0.78/0.89/0.99 |
| (*E*)*-*β-ocimene | 0.15 | ± | 0.15 | 1.00 | ± | 0.63 |  | 12.05 | ± | 10.34 | 1.56 | ± | 0.54 |  | 0.800 | **0.051** | 0.246 |  | 0.92/**1.01**/0.90 |
| α-terpinolene | 0.56 | ± | 0.56 | - |  |  |  | 0.62 | ± | 0.62 | - |  |  |  | 0.173 | 0.971 | 0.971 |  | 0.56/0.65/0.58 |
| linalool | 0.46 | ± | 0.46 | 0.34 | ± | 0.34 |  | 1.18 | ± | 1.18 | 0.62 | ± | 0.62 |  | 0.813 | 0.701 | 0.917 |  | 0.24/0.19/0.19 |
| Unknown monoterpene† | 2.38 | ± | 0.62 | 1.39 | ± | 0.37 |  | 3.21 | ± | 0.66 | 1.04 | ± | 0.36 |  | **0.012** | 0.793 | 0.228 |  | **1.32**/**1.14**/**1.02** |
| (*E*)*-*β*-*caryophylene | - |  |  | 0.24 | ± | 0.11 |  | 0.50 | ± | 0.14 | 0.20 | ± | 0.15 |  | 0.790 | **0.061** | **0.022** |  | **1.10**/**1.13**/**1.12** |
| Total Terpenoids | 33.58 | ± | 7.02 | 20.11 | ± | 4.53 |  | 72.46 | ± | 19.88 | 28.73 | ± | 6.08 |  | **0.007** | **0.040** | 0.504 |  |  |
| Green leaf volatiles (GLV) |  |  |  |  |  |  |  |  |  |  |  |  |  |  |  |  |  |  |  |
| (*Z*)-3-hexen-1-ol | 3.49 | ± | 1.75 | 1.17 | ± | 0.69 |  | 4.21 | ± | 1.61 | - |  |  |  | **0.020** | 0.677 | 0.241 |  | **1.01**/**1.09**/**1.01** |
| (*Z*)-3-hexen-1-ol acetate | 132.31 | ± | 65.70 | 34.17 | ± | 9.21 |  | 59.53 | ± | 28.51 | 4.69 | ± | 1.46 |  | **0.030** | **0.068** | 0.188 |  | 0.56/**1.26**/**1.35** |
| Total GLV | 135.80 | ± | 67.37 | 35.35 | ± | 9.80 |  | 63.74 | ± | 29.81 | 4.69 | ± | 1.46 |  | **0.013** | 0.669 | **0.092** |  |  |
| Other compounds |  |  |  |  |  |  |  |  |  |  |  |  |  |  |  |  |  |  |  |
| 1-octen-3-ol | - |  |  | - |  |  |  | 45.44 | ± | 12.05 | 8.44 | ± | 4.09 |  | **0.003** | <**0.001** | **0.003** |  | **2.02**/**1.81**/**1.67** |
| methyl salicylate | 1.64 | ± | 0.92 | 0.60 | ± | 0.43 |  | 1.11 | ± | 0.55 | 0.44 | ± | 0.44 |  | 0.193 | 0.671 | 1.000 |  | 0.39/0.74/0.65 |
| Total VOCs | 171.05 | ± | 67.55 | 56.50 | ± | 13.33 |  | 183.68 | ± | 37.83 | 42.33 | ± | 4.44 |  | **0.003** | 0.578 | 0.343 |  |  |

ǂ Bold numbers indicate significant or marginally significant effects of endophyte (E), aphid (A) or their interaction (E×A) as determined by individual two-way ANOVAs based on log-transformed data. Numbers within the brackets denote sample size.

§ Variable Importance in the Projection (VIP) scores for PLS-DA are given for the first three components, which are separated by slashes. VIP scores highlighted in bold are higher than 1 and are most influential for separation of individual treatments.

† Compounds are tentatively identified.
