# Supplementary material for: Epichloë Endophytes Alter Inducible Indirect Defences in Host Grasses
Source: PLoS One. 2014 Jun 30;9(6):e101331. doi: 10.1371/journal.pone.0101331 (PMC4076332; doi:10.1371/journal.pone.0101331)
Supplement: Table S6 — VOC emissions (ng gDW-1 h-1) from meadow fescue at 6 days post damage. E-: naturally endophyte free; E+: naturally endophyte infected. (DOCX) [file pone.0101331.s011.docx]

Table S6. VOC emissions (ng gDW^-1^ h^-1^) from meadow fescue at 6 days post damage. E-: naturally endophyte free; E+: naturally endophyte infected.

|  | Control | | | | | |  | Aphid | | | | | |  | Wounding | | | | | |  | *P*ǂ | | |  | VIP scores§ |
| --- | --- | --- | --- | --- | --- | --- | --- | --- | --- | --- | --- | --- | --- | --- | --- | --- | --- | --- | --- | --- | --- | --- | --- | --- | --- | --- |
| Compound | E- (6) | | | E+ (6) | | |  | E- (6) | | | E+ (6) | | |  | E- (6) | | | E+ (6) | | |  | E | D | E**×**D |  |  |
| Terpenoids |  |  |  |  |  |  |  |  |  |  |  |  |  |  |  |  |  |  |  |  |  |  |  |  |  |  |
| α-pinene | 3.96 | ± | 0.60 | 4.51 | ± | 1.05 |  | 4.34 | ± | 0.64 | 4.99 | ± | 1.39 |  | 4.15 | ± | 0.79 | 4.14 | ± | 0.86 |  | 0.910 | 0.871 | 0.966 |  | 0.02/0.27 |
| 6-methyl-5-hepten-2-one† | 0.21 | ± | 0.13 | 0.11 | ± | 0.11 |  | 0.29 | ± | 0.20 | 0.09 | ± | 0.07 |  | 0.36 | ± | 0.21 | - |  |  |  | **0.065** | 0.983 | 0.634 |  | 0.85/0.68 |
| β-myrcene | 13.01 | ± | 1.83 | 6.74 | ± | 1.66 |  | 18.85 | ± | 3.79 | 11.19 | ± | 2.26 |  | 17.60 | ± | 4.41 | 6.36 | ± | 2.38 |  | **0.000** | 0.110 | 0.441 |  | **1.57/1.35** |
| β-pinene | 0.70 | ± | 0.06 | 0.76 | ± | 0.15 |  | 0.75 | ± | 0.07 | 0.81 | ± | 0.14 |  | 0.69 | ± | 0.07 | 0.64 | ± | 0.13 |  | 0.990 | 0.559 | 0.834 |  | 0.12/0.48 |
| δ-carene | 0.69 | ± | 0.20 | 0.89 | ± | 0.27 |  | 0.86 | ± | 0.22 | 0.92 | ± | 0.15 |  | 0.65 | ± | 0.16 | 0.75 | ± | 0.23 |  | 0.560 | 0.648 | 0.973 |  | 0.15/0.47 |
| (*Z*)-β-ocimene† | 0.76 | ± | 0.27 | 0.90 | ± | 0.44 |  | 1.82 | ± | 0.37 | 2.73 | ± | 1.09 |  | 1.89 | ± | 0.73 | 1.49 | ± | 0.96 |  | 0.699 | 0.147 | 0.775 |  | 0.40/0.90 |
| d-limonene | 6.72 | ± | 1.34 | 2.35 | ± | 1.02 |  | 10.02 | ± | 2.48 | 5.64 | ± | 1.67 |  | 9.50 | ± | 2.82 | 2.69 | ± | 1.50 |  | <**0.001** | 0.115 | 0.467 |  | **1.53**/**1.33** |
| β-phellandrene† | 1.62 | ± | 0.25 | 0.79 | ± | 0.30 |  | 2.25 | ± | 0.37 | 0.79 | ± | 0.51 |  | 2.21 | ± | 0.44 | 0.61 | ± | 0.34 |  | <**0.001** | 0.948 | 0.584 |  | **1.54**/**1.29** |
| (*E*)*-*β-ocimene | 0.36 | ± | 0.36 | 0.69 | ± | 0.34 |  | 0.59 | ± | 0.37 | 2.26 | ± | 1.02 |  | 0.64 | ± | 0.48 | 0.80 | ± | 0.57 |  | 0.208 | 0.497 | 0.668 |  | 0.35/0.92 |
| α-terpinolene | 1.73 | ± | 0.21 | 0.47 | ± | 0.30 |  | 2.36 | ± | 0.49 | 1.20 | ± | 0.42 |  | 2.07 | ± | 0.56 | 0.57 | ± | 0.40 |  | **0.000** | 0.255 | 0.822 |  | **1.50**/**1.22** |
| linalool | 1.31 | ± | 0.28 | 0.94 | ± | 0.24 |  | 2.57 | ± | 0.72 | 1.41 | ± | 0.94 |  | 2.57 | ± | 0.27 | 0.65 | ± | 0.22 |  | **0.001** | 0.477 | 0.197 |  | **1.43**/**1.13** |
| Unknown monoterpene† | 2.65 | ± | 0.47 | 1.39 | ± | 0.44 |  | 2.99 | ± | 0.45 | 1.98 | ± | 0.34 |  | 3.25 | ± | 0.40 | 1.06 | ± | 0.22 |  | <**0.001** | 0.365 | 0.289 |  | **1.60**/**1.30** |
| (*E*)*-*β*-*caryophylene | 0.07 | ± | 0.07 | 0.20 | ± | 0.13 |  | 0.16 | ± | 0.16 | 0.57 | ± | 0.38 |  | 0.32 | ± | 0.16 | 0.06 | ± | 0.06 |  | 0.624 | 0.566 | 0.183 |  | 0.05/0.87 |
| Total Terpenoids | 33.58 | ± | 4.18 | 20.62 | ± | 4.22 |  | 47.55 | ± | 6.51 | 34.51 | ± | 4.12 |  | 45.55 | ± | 9.33 | 19.81 | ± | 4.62 |  | **<0.001** | **0.029** | 0.322 |  |  |
| Green leaf volatiles (GLV) |  |  |  |  |  |  |  |  |  |  |  |  |  |  |  |  |  |  |  |  |  |  |  |  |  |  |
| (*Z*)-3-hexen-1-ol | 1.61 | ± | 0.60 | 2.00 | ± | 1.02 |  | 2.70 | ± | 1.31 | - |  |  |  | 3.64 | ± | 0.89 | 0.98 | ± | 0.64 |  | **0.011** | 0.238 | 0.196 |  | 0.95/**1.25** |
| (*Z*)-3-hexen-1-ol acetate | 42.85 | ± | 7.30 | 44.19 | ± | 19.21 |  | 74.07 | ± | 44.03 | 5.49 | ± | 1.55 |  | 62.22 | ± | 27.91 | 21.06 | ± | 9.23 |  | **0.008** | 0.147 | 0.525 |  | 0.85/**1.41** |
| Total GLV | 44.46 | ± | 7.45 | 46.19 | ± | 20.22 |  | 76.77 | ± | 45.18 | 5.49 | ± | 1.55 |  | 65.86 | ± | 28.49 | 22.04 | ± | 9.84 |  | **0.006** | 0.138 | 0.502 |  |  |
| Other compounds |  |  |  |  |  |  |  |  |  |  |  |  |  |  |  |  |  |  |  |  |  |  |  |  |  |  |
| 1-octen-3-ol | - |  |  | - |  |  |  | 1.10 | ± | 1.10 | - |  |  |  | - |  |  | - |  |  |  | 0.325 | 0.380 | 0.380 |  | 0.49/0.42 |
| methyl salicylate | 2.15 | ± | 0.93 | 0.82 | ± | 0.82 |  | 1.33 | ± | 0.67 | 1.56 | ± | 1.56 |  | 2.10 | ± | 1.65 | 2.02 | ± | 0.89 |  | 0.441 | 0.753 | 0.494 |  | 0.16/0.42 |
| Total VOCs | 80.40 | ± | 11.50 | 67.74 | ± | 19.07 |  | 127.05 | ± | 46.52 | 41.64 | ± | 4.62 |  | 113.88 | ± | 29.37 | 43.87 | ± | 8.78 |  | **0.001** | 0.967 | 0.391 |  |  |

ǂ Bold numbers indicate significant or marginally significant effects of endophyte (E), damage treatment (D) or their interaction (E×D) as determined by individual two-way ANOVAs based on log-transformed data. Numbers within the brackets denote sample size.

§ Variable Importance in the Projection (VIP) scores for PLS-DA are given for the first three components, which are separated by slashes. VIP scores highlighted in bold are higher than 1 and are most influential for separation of individual treatments.

† Compounds are tentatively identified.
