# Supplementary material for: Epichloë Endophytes Alter Inducible Indirect Defences in Host Grasses
Source: PLoS One. 2014 Jun 30;9(6):e101331. doi: 10.1371/journal.pone.0101331 (PMC4076332; doi:10.1371/journal.pone.0101331)
Supplement: Table S8 — VOC emissions (ng gDW-1 h-1) from meadow fescue at 1 day after mechanical wounding. E-: naturally endophyte free; E+: naturally endophyte infected. (DOCX) [file pone.0101331.s013.docx]

Table S8. VOC emissions (ng gDW^-1^ h^-1^) from meadow fescue at 1 day after mechanical wounding. E-: naturally endophyte free; E+: naturally endophyte infected.

|  | Control | | | | | |  | Wounding | | | | | |  | *P*ǂ | | |  | VIP scores§ |
| --- | --- | --- | --- | --- | --- | --- | --- | --- | --- | --- | --- | --- | --- | --- | --- | --- | --- | --- | --- |
| Compound | E- (6) | | | E+ (6) | | |  | E- (6) | | | E+ (6) | | |  | E | W | ExW |  |  |
| Terpenoids |  |  |  |  |  |  |  |  |  |  |  |  |  |  |  |  |  |  |  |
| α-pinene | 0.98 | ± | 0.21 | 0.77 | ± | 0.22 |  | 0.98 | ± | 0.23 | 0.44 | ± | 0.11 |  | 0.064 | 0.399 | 0.408 |  | **1.02**/**1.03** |
| 6-methyl-5-hepten-2-one† | 0.41 | ± | 0.19 | 0.08 | ± | 0.08 |  | 0.42 | ± | 0.11 | 0.10 | ± | 0.10 |  | **0.010** | 0.784 | 0.918 |  | **1.19**/**1.08** |
| β-myrcene | 19.40 | ± | 3.71 | 8.74 | ± | 2.59 |  | 21.90 | ± | 4.33 | 8.16 | ± | 2.78 |  | **0.002** | 0.974 | 0.705 |  | **1.45**/**1.16** |
| β-pinene | 0.31 | ± | 0.06 | 0.26 | ± | 0.06 |  | 0.32 | ± | 0.07 | 0.14 | ± | 0.04 |  | **0.056** | 0.266 | 0.256 |  | **1.08**/**1.18** |
| δ-carene | 0.32 | ± | 0.10 | 0.42 | ± | 0.13 |  | 0.29 | ± | 0.09 | 0.18 | ± | 0.09 |  | 0.907 | 0.197 | 0.326 |  | 0.27/**1.56** |
| (*Z*)-β-ocimene† | 1.62 | ± | 0.47 | 1.35 | ± | 0.60 |  | 2.51 | ± | 1.14 | 3.21 | ± | 1.77 |  | 0.809 | 0.347 | 0.706 |  | 0.01/0.86 |
| d-limonene | 17.29 | ± | 2.86 | 10.55 | ± | 2.25 |  | 19.15 | ± | 4.06 | 8.18 | ± | 2.06 |  | **0.005** | 0.679 | 0.465 |  | **1.38**/**1.03** |
| β-phellandrene† | 2.35 | ± | 0.37 | 1.16 | ± | 0.30 |  | 2.48 | ± | 0.37 | 0.79 | ± | 0.33 |  | **0.001** | 0.574 | 0.396 |  | **1.57**/**1.17** |
| (*E*)*-*β-ocimene | 0.48 | ± | 0.35 | 0.71 | ± | 0.49 |  | 1.37 | ± | 0.94 | 1.81 | ± | 1.02 |  | 0.641 | 0.231 | 0.859 |  | 0.31/0.83 |
| α-terpinolene | 2.30 | ± | 0.55 | 0.82 | ± | 0.42 |  | 2.68 | ± | 0.69 | 0.79 | ± | 0.39 |  | **0.009** | 0.868 | 0.841 |  | **1.22**/0.98 |
| linalool | 2.70 | ± | 0.50 | 2.23 | ± | 0.36 |  | 5.67 | ± | 1.47 | 2.07 | ± | 0.60 |  | **0.028** | 0.320 | 0.100 |  | **1.16**/**1.17** |
| Unknown monoterpene† | 3.10 | ± | 0.82 | 2.67 | ± | 1.08 |  | 4.07 | ± | 1.08 | 0.91 | ± | 0.41 |  | **0.035** | 0.651 | 0.176 |  | **1.18**/0.98 |
| (*E*)*-*β*-*caryophylene | 0.13 | ± | 0.13 | 0.09 | ± | 0.09 |  | 0.42 | ± | 0.26 | 0.09 | ± | 0.09 |  | 0.336 | 0.443 | 0.426 |  | 0.56/0.75 |
| Total Terpenoids | 50.99 | ± | 8.38 | 29.77 | ± | 7.17 |  | 61.84 | ± | 13.35 | 26.76 | ± | 5.66 |  | **0.004** | 0.778 | 0.630 |  |  |
| Green leaf volatiles (GLV) |  |  |  |  |  |  |  |  |  |  |  |  |  |  |  |  |  |  |  |
| (*Z*)-3-hexen-1-ol | 1.83 | ± | 0.87 | 1.01 | ± | 0.66 |  | 1.54 | ± | 0.52 | 0.61 | ± | 0.39 |  | 0.205 | 0.884 | 0.799 |  | 0.67/0.50 |
| (*Z*)-3-hexen-1-ol acetate | 37.41 | ± | 19.45 | 14.65 | ± | 6.94 |  | 16.95 | ± | 3.99 | 11.69 | ± | 3.51 |  | 0.223 | 0.612 | 0.876 |  | 0.59/0.45 |
| Total GLV | 39.23 | ± | 20.11 | 15.65 | ± | 7.49 |  | 18.48 | ± | 4.34 | 12.30 | ± | 3.79 |  | 0.224 | 0.641 | 0.927 |  |  |
| Other compounds |  |  |  |  |  |  |  |  |  |  |  |  |  |  |  |  |  |  |  |
| 1-octen-3-ol | - |  |  | - |  |  |  | - |  |  | - |  |  |  |  |  |  |  |  |
| methyl salicylate | 2.50 | ± | 1.52 | 5.93 | ± | 2.92 |  | 4.94 | ± | 1.51 | 1.31 | ± | 0.97 |  | 0.451 | 0.968 | **0.053** |  | 0.64/0.67 |
| Total VOCs | 93.13 | ± | 24.60 | 51.44 | ± | 14.56 |  | 85.68 | ± | 12.96 | 40.46 | ± | 6.36 |  | **0.008** | 0.920 | 0.778 |  |  |

ǂ Bold numbers indicate significant or marginally significant effects of endophyte (E), wounding (W) or their interaction (E×W) as determined by individual two-way ANOVAs based on log-transformed data. Numbers within the brackets denote sample size.

§ Variable Importance in the Projection (VIP) scores for PLS-DA are given for the first three components, which are separated by slashes. VIP scores highlighted in bold are higher than 1 and are most influential for separation of individual treatments.

† Compounds are tentatively identified.
